# A Potential Checkmate to Lead: Bismuth in Organometal Halide Perovskites, Structure, Properties, and Applications

**DOI:** 10.1002/advs.201903143

**Published:** 2020-05-27

**Authors:** Sanam Attique, Nasir Ali, Shahid Ali, Rabia Khatoon, Na Li, Amir Khesro, Sajid Rauf, Shikuan Yang, Huizhen Wu

**Affiliations:** ^1^ Institute for Composites Science and Innovation (InCSI) School of Material Science and Engineering Zhejiang University Hangzhou 310027 P. R. China; ^2^ Zhejiang Province Key Laboratory of Quantum Technology and Devices and Department of Physics State Key Laboratory for Silicon Materials Zhejiang University Hangzhou 310027 P. R. China; ^3^ Materials Research Laboratory Department of Physics University of Peshawar Peshawar 25120 Pakistan; ^4^ State Key Laboratory of Silicon Materials School of Materials Science and Engineering Zhejiang University Hangzhou 310027 P. R. China; ^5^ Department of Chemistry and Chemical Engineering School of Chemistry and Biological Engineering University of Science and Technology Beijing Beijing 100083 P. R. China; ^6^ Department of Physics Abdul Wali Khan University Mardan 23200 Pakistan; ^7^ Hubei Collaborative Innovation Centre for Advanced Organic Chemical Materials Faculty of Physics and Electronic Science Hubei University Wuhan Hubei 430062 P. R. China

**Keywords:** bismuth‐based perovskites, lead‐free perovskites, optoelectronic properties, optoelectronics, structural properties

## Abstract

The remarkable optoelectronic properties and considerable performance of the organo lead‐halide perovskites (PVKs) in various optoelectronic applications grasp tremendous scientific attention. However, the existence of the toxic lead in these compounds is threatening human health and remains a major concern in the way of their commercialization. To address this issue, numerous nontoxic alternatives have been reported. Among these alternatives, bismuth‐based PVKs have emerged as a promising substitute because of similar optoelectronic properties and extended environmental stability. This work communicates briefly about the possible lead‐alternatives and explores bismuth‐based perovskites comprehensively, in terms of their structures, optoelectronic properties, and applications. A brief description of lead‐toxification is provided and the possible Pb‐alternatives from the periodic table are scrutinized. Then, the classification and crystal structures of various Bi‐based perovskites are elaborated on. Detailed optoelectronic properties of Bi‐based perovskites are also described and their optoelectronic applications are abridged. The overall photovoltaic applications along with device characteristics (i.e., *V*_OC_, *J*_SC_, fill factor, FF, and power conversion efficiency, PCE), fabrication method, device architecture, and operational stability are also summarized. Finally, a conclusion is drawn where a brief outlook highlights the challenges that hamper the future progress of Bi‐based optoelectronic devices and suggestions for future directions are provided.

## Graphical abstract

The toxic‐lead in organometal‐halide perovskites is hindering their applications and readily needs a replacement. This work communicates briefly about the possible nontoxic lead alternatives and explores bismuth‐based perovskites comprehensively, in terms of their structures, optoelectronic properties, and applications. This work is helpful to improve the material and performance of the bismuth‐based perovskites to bring them on par with lead‐based perovskites.

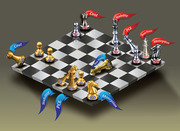

## 1 Introduction

Owing to their enormous optical and electronic properties along with facile solution processability, organometal‐halide perovskite (PVKs) captivated the scientific community.^[^ ^1^ ^]^ PVK materials are preferred because of their defect tolerant nature, significantly larger absorption coefficient, extended carrier lifetime, tunable bandgap, lower exciton energy, and excellent light‐harvesting and emitting properties.^[^ ^2^ ^]^ Still, perovskite solar‐cells (PSCs) hold the center of attention in the photovoltaic market by having the power conversion efficiency (PCE) that exceeds over 25%, making it a rival to the conventional silicon‐based solar‐cells.^[^ ^3^ ^]^

Besides such peculiar advantages, many pitfalls are associated with PVK materials. For instance, they are extremely unstable against environmental factors, i.e., moisture ingress, thermal stress, and ultraviolet (UV) radiations. Moreover, organic–inorganic lead‐halide PVKs ride on the toxic bioaccumulative lead (Pb), which ultimately hinders its industrialization. Currently, the issue of moisture ingression is addressed partially by proper encapsulation of a device, surface passivation, and the combination of lower‐dimensional PVKs (e.g., 2D PVKs) with the bulk PVKs structures.^[^ ^4^ ^]^ Similarly, the vulnerability of PVKs to UV‐light could successfully be resolved by the down conversion, initiated by the metallic nanoparticles introduced into the device structure.^[^ ^5^ ^]^ Likewise, the issue of thermal decomposition could be dealt with the employment of 2D materials (e.g., graphene and its derivatives) on the top of the active PVK layer.^[^ ^6^ ^]^ Herein, our discussion will be limited to the toxicity of lead in organometal‐halide PVKs and their solution by lead‐free PVKs. For the detailed study of the decomposition in organometal‐halide PVKs, readers are directed to our previous review paper.^[^ ^4a^ ^]^

To address the issue of toxicity, researchers are struggling to find environment‐friendly Pb‐free alternatives. In this regard, many materials have been introduced into the PVK‐structure; for instance, Sn‐based PVKs were introduced and were found to be more pronounced due to their excellent performance in solar‐cells with a considerable PCE of 12.04%.^[^ ^7^ ^]^ Unfortunately, Sn‐based PVKs were extremely unstable, i.e., oxidized rapidly from Sn^2+^ to Sn^4+^, thereby formed a completely different non‐PVK phase (i.e., A_2_SnX_4_).^[^ ^8^ ^]^

Beside Sn, many other divalent and trivalent alternatives, e.g., Ge^2+^, Mg^2+^, Mn^2+^_,_ Bi^3+^, Sb^3+^, etc. were also introduced.^[^ ^9^ ^]^ Herein, our discussion will be limited to the trivalent bismuth (i.e., Bi^3+^), which is expected to be the best replacement for Pb^2+^ (in the iconic organometal lead‐halide PVKs) due to the following advantages: i) outstanding environmental stability,^[^ ^10^ ^]^ ii) greater absorption coefficient (≈10^5^ cm^−1^),^[^ ^11^ ^]^ iii. similar ionic radius to Pb^2+^,^[^ ^12^ ^]^ and iv) isoelectronicity with Pb (presence of 6s^2^electrons).^[^ ^12^, ^13^ ^]^ Moreover, due to their octahedral‐coordinated structure (one class of the defect‐tolerant materials), a longer carrier diffusion lifetime is expected from Bi‐based PVKs.^[^ ^14^ ^]^ These remarkable properties enabling Bi as a promising candidate to substitute the toxic Pb in the PVKs framework.^[^ ^15^ ^]^

After the pioneering work on Bi‐based PVK solar‐cells by Park et al., it has been widely utilized in the PVK structures and exercised extensively for many optoelectronic applications.^[^ ^16^ ^]^ In the first section of this progress report, the toxicity of Pb and its lethal effects on the human body as well as the possible Pb‐alternatives including their advantages and drawbacks are discussed. The second portion is related to the structural details about the various classes (based on crystal symmetry) of the Bi‐based PVKs are provided. The third and fourth sections are devoted to the overall optoelectronic properties and applications of the Bi‐based PVKs, respectively. Finally, a brief outlook in terms of challenges remaining in the way of further development of the Bi‐based PVKs and our remarks to resolve these issues and step forward for its commercialization are given.

## 2 Lead‐Free PVKs

**Figure 1:**
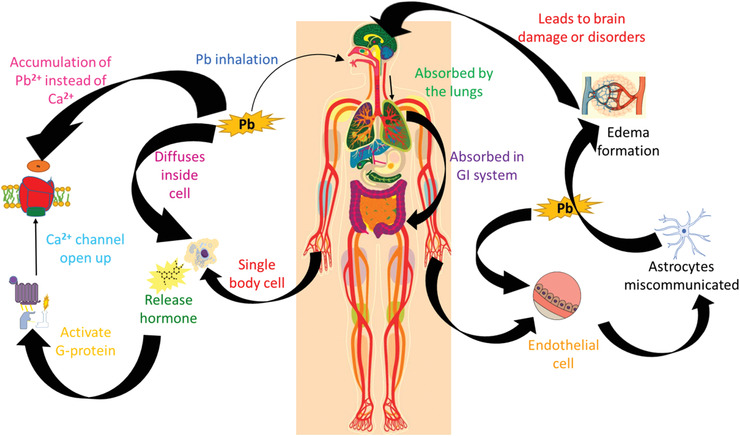
Illustration of the lead toxification in the human body: at the cellular (left) and neurological (right) levels.

Lead, as an essential element in the organometal halide PVKs has raised the concern of toxicity, which dwells to damage the ecosystem as the only level of lead exposure not detrimental to human health is zero. The solubility constant of lead in water, i.e., *K*_sp_ = 4.4 × 10^−9^ and even no safe threshold level of lead in blood is mentioned.^[^ ^17^ ^]^ Therefore, without proper encapsulation, rainwater can simply decompose the PVK‐structure and leaches the Pb down from the rooftop energy harvesting devices (e.g., solar‐panels) that can easily contaminate the environment. Besides this, the toxic Pb used in such optoelectronic devices cannot be recycled as in the case of other energy harvesting devices. Lead particles when inhaled, are absorbed by the lungs and some of them are taken to the throat during mucociliary clearance where they are absorbed by the gastrointestinal system.^[^ ^18^ ^]^ Once the body has been disrupted by lead, there is no viable way to undo it. Lead ions, when diffused inside the blood can cause damage not only at the organ level but also at the cellular level, as summarized in **Figure** **1**.^[^ ^18^, ^19^ ^]^ In fact, the existence of lead in optoelectronic devices, which are exposed to the environment is an alarming threat to all living beings. Therefore, besides its tremendous advantages and excellent performance in the optoelectronic devices; for instance, higher PCE in PVKs solar‐cells, it readily needs a suitable substitute that is nontoxic at least lesser toxic than lead. This new urge triggered a novel research field based on lead‐free metal halide PVKs.

**Figure 2:**
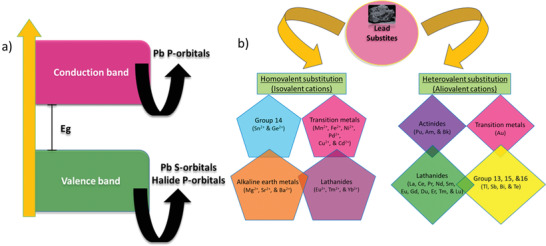
a) Energy band diagram for conventional Pb‐based organometal halide PVKs. b) List of the nontoxic lead substitutes.

**Figure 3:**
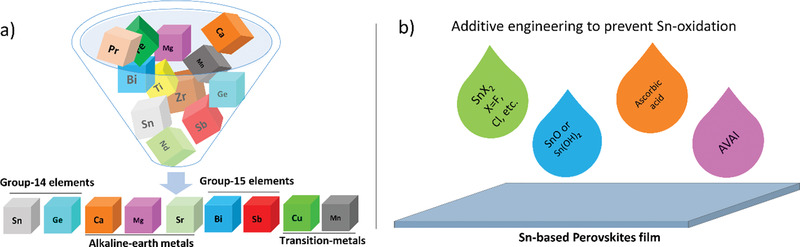
a) Possible non‐ or less‐toxic alternatives to replace Pb (filtered from the periodic table) in the organometal halide perovskite framework. b) Additive engineering to maintain Sn in its bivalent state and improve the film morphology. Various species are shown which are utilized into the precursor solution of the Sn‐based perovskites.

While replacing Pb, the electronic configuration of the typical lead‐halide PVKs should be taken into account where the valence band is made up of halide's p‐orbitals and lead's s‐orbitals, while conduction band is solely based on lead's p‐orbitals, as shown in **Figure** **2**a.^[^ ^20^ ^]^ The elements imitating this electronic structure or at least following the order of the Pb electronic configuration are therefore preferred as potential alternatives. Consequently, elements with filled 6s‐orbitals (to make up the valence band) and almost empty p‐orbital (to fill up the conduction band) are inevitable to replace Pb in the PVKs framework. In this regard, numerous elements are in the list, which acquires the same or maybe better electronic properties than Pb. One way is to simply move up‐down or left‐right in or from the same group that belongs to Pb and figure out the best alternative in terms of similar electronic configuration. In Figure 2b we have summarized all the elements that could possibly substitute the toxic Pb in the PVK architecture. These elements were scrutinized and it was found that some of them should be eliminated from the list due to the following reasons: i) finite competence to form PVKs, ii) some are not appropriate for photovoltaic (PV) applications, as they acquire much higher bandgaps, iii) some are ruled out because of their higher toxicity even greater than Pb, and iv) some are omitted due to their environmental vulnerabilities.^[^ ^12^, ^21^ ^]^ Keeping in view the above considerations, the promising candidates for Pb substitution are summarized, as shown in **Figure** **3**a, which will be further discussed in the next section.

### 2.1 Group 14 Elements (Sn and Ge)

Tin (Sn) and Germanium (Ge) are the ultimate first‐class choice while looking for Pb‐free alternatives as they are more active and comparatively less toxic.^[^ ^22^ ^]^ Therefore, both of these alternatives remained under the spotlight of the scientific community. Unfortunately, due to poor performance in the optoelectronic devices (e.g., lower PCE in solar cells), Ge is less preferred as compared to Sn. Moreover, Ge‐based PVKs exhibited poor stability due to their oxidation from Ge^2+^ to Ge^4+^. The oxidation issue in Ge‐based PVKs was found to be even more severe than the Sn^2+^ because of the reduced inert electron pair, which also lead toward lower dimensionality and significantly reduced conductivities. Additionally, their smaller ionic radii, as well as distortion of its octahedron [GeX_6_]^3−^, eventually became the cause of a slightly high bandgap > 1.6 eV, which could have also contributed to its poor performance in PSCs. Owing to their lower enthalpies, Ge‐based PVKs also exhibited a greater defect formation, e.g., significantly high hole‐density.^[^ ^23^ ^]^ Therefore, regardless of their nontoxic nature, its defect chemistry and rapid oxidation restricted the PCE of Ge‐based solar‐cells below 5% and hindered their applications in photovoltaics.^[^ ^24^ ^]^

Similarly, Sn‐based PVKs got more attention by having several distinguished properties; including direct bandgap, lower exciton binding energy, superior carrier mobility, etc.^[^ ^25^ ^]^ Despite enormous efforts, Sn‐based PVKs could gain only about 12% of PCE, which could not be retained for longer due to its unwanted oxidation from Sn^2+^ to Sn^4+^ in the air.^[^ ^7^, ^26^ ^]^ Thus, resulted in poor reproducibility and eventually lead to the destruction of a device. Various strategies have been applied to overcome the issue of the oxidation state conversion; for instance, many additives have been added into the precursor solution of the Sn‐based PVKs, which unfortunately failed to stabilize the Sn in its bivalent form, see Figure 3b.^[^ ^27^ ^]^

### 2.2 Alkaline Earth Metals PVKs

Alkaline earth metals being earth‐abundant metals also share the same oxidation state as that of lead; therefore, candidates from this group (e.g., Mg^2+^, Ca^2+^, Sr^2+^, and Ba^2+^) serve as another option to substitute the toxic Pb. Like, Sn‐ and Ge‐based PVKs, alkaline earth metals also form the same 3D PVKs structure, i.e., ABX_3_; where, A = (methylammonium or MA^+^, formamidinium or FA^+^, Cs^+^, K^+^, etc.), B = (Mg^2+^, Ca^2+^, Sr^2+^, etc.), and X = (Cl^−^, Br^−^, or I^−^). They are nontoxic and exhibited better photoluminescence properties when doped with rare earth metals.^[^ ^28^ ^]^ Unfortunately, due to their higher bandgaps and environmental vulnerabilities, they also failed to deliver the desired device performance.

### 2.3 Transition Metal Halides PVKs

Transition metals are found to be environmentally stable and their 2D‐layered structure made them favorable for more cation sites variations. In other words, they allowed a variety of organic cations due to their 2D body‐centered structure.^[^ ^29^ ^]^ Although some preliminary theoretical work and experimental results were carried out for these materials (e.g., mixed halide Cu‐based PVKs) but no extraordinary progress regarding their device performance has been reported.^[^ ^29^, ^30^ ^]^

### 2.4 Ferroelectric PVKs Material

Ferroelectric type materials were previously exploited for their magnetic properties but somehow it was also suggested that they could also be used as photoabsorbers.^[^ ^31^ ^]^ In this regard, La_2_NiMnO_6_ PVK compound was fabricated;^[^ ^32^ ^]^ however, its complicated fabrication process (i.e., pulsed laser and physical vapor deposition) made it extremely expensive and thus hindered its further reproducibility.

From the above brief discussion, it can be recapitulated that up till now Sn and Ge are presumed to be relatively better options in terms of considerable PCE and less toxicity. However, Ge‐based PVKs analogs severely underperformed in PV devices; the reasons behind were, i) its smaller ionic radius, ii) distortion of their octahedron [GeX_6_]^3‐^ that eventually lead to a wider bandgap (>1.6), iii) unwanted oxidation, i.e., from Ge^2+^into Ge^4+^, and iv) poor solubility of their compounds in the organic solvents, resulting in an inappropriate morphology. These problems consequently deteriorated the device performance, e.g., yielded a lower PCE ≈0.2% with poor stability.^[^ ^33^ ^]^

Similarly, the most popular among Pb‐free PVKs, i.e., Sn‐based PVKs possessed many promising properties, e.g., high carrier mobility, narrow bandgap, and similar crystal structure as Pb‐based PVKs. Albeit to these extraordinary similarities and considerable photovoltaic (PV) performance, Sn‐based PVKs encountered poor stability initiated by its self‐doping from Sn^2+^ to Sn^4+^. Various additives via solvent‐engineering have been exploited to retain it in its bivalent form, but that could only be succeeded for a very short time. Still, the issue of instability in Ge and Sn‐based PVKs remained an open challenge. Moreover, Sn is also been reported as a toxic element.^[^ ^34^ ^]^ Thus if not Sn, then what next? This urge took the PVK community to the next level and they focused on group‐15 elements, specifically, antimony (Sb) and Bi. These elements were far less toxic than Pb and showed similar electronic structure, i.e., filled s‐orbitals and pretty much empty p‐orbital. Most importantly, they were proven to be more stable (when utilized in PVK structures) than other Pb‐free options. Herein, our discussion will be limited to Bi‐based PVKs only.

## 3 Bi‐Based PVKs

Bismuth, being a member of group‐15 of the periodic table shares similar electronegativity, electronic configuration, and a comparable ionic radius with lead. As far as toxicity is concerned, Bi is less toxic than Pb and is found abundantly in the earth's crust, which makes it cost‐effective as well. Among many Pb analogs, Bi is a promising option because of its exceptional air stability, better optophysical properties, and excellent defect tolerance capability.^[^ ^35^ ^]^ Having similar active ns^2^ electronic configuration to that of Pb, which tends to build up an antibonding interaction at valence band maximum (VBM), leading to the confinement of defects to the shallow states at energy band edges. Thus, regardless of having defect sites, Bi has the potential to retain its optoelectronic properties.^[^ ^36^ ^]^ Being an aliovalent metal cation, Bi has different oxidation states compared to Pb, which makes it difficult to attain charge neutrality in the typical ABX_3_PVK structure.^[^ ^37^ ^]^ This necessitates the need to balance the charge between B‐site cations and X‐site halide anions. One way to deal with it is a mixed valency approach, where monovalent and trivalent cations are replaced to establish an overall divalent state.^[^ ^38^ ^]^ For Bi‐based PVKs, a single corner‐sharing among metal halide octahedra is not common because their lattices are comprised of distorted MX_6_ octahedra that buildup a mono‐nuclear or poly‐nuclear network of corner, edge, or face shared octahedra.^[^ ^20b^ ^]^ Due to this structural diversity, Bi‐based PVKs can be categorized as zero (dimer units), one (chain‐like motifs), two, and three (elpasolites) dimensional structures, as summarized below.^[^ ^39^ ^]^

### 3.1 0D Bi‐Based PVKs

**Figure 4:**
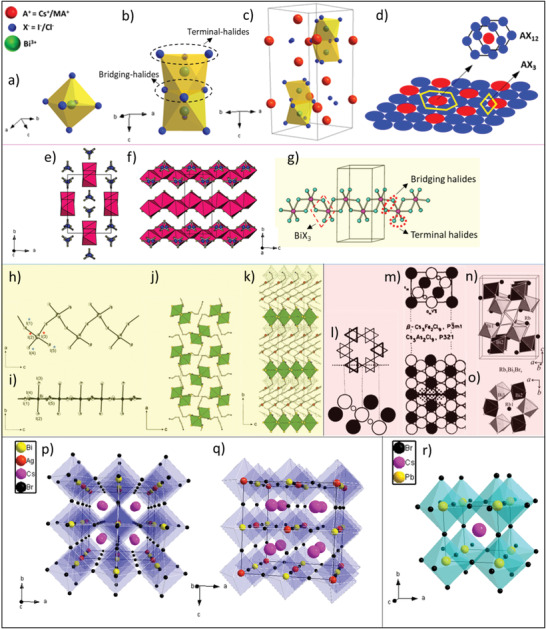
Crystallographic structures of various families of the Bi‐based perovskites: a,b) BiX_6_ octahedron and Bi_2_X_9_ bioctahedra. c) Crystal structure of the 0D A_3_Bi_2_X_9_ perovskite. d) Visualization of the hexagonal closed packed structure formed by the combination of AX_3_. e,f) The crystal structure of LiBiI_4_·5H_2_O viewed along the “*c*” and “*a*” axis, respectively. Reproduced with permission.^[^ ^44^ ^]^ Copyright 2016, American Chemical Society. g) A fragment of 1D (BiI_4_)^−^ chain. h,i) The BiI_5_^2−^ chain structure for (H_2_AETH)BiI_5_: top and side views. Reproduced with permission.^[^ ^44^, ^45^ ^]^ Copyright 2001, 2016, American Chemical Society. j) Crystal structure of (H_2_DAH)BiI_5_: viewed down an axis, i.e., parallel to the BiI_5_^2−^ chains. k) Crystal structure of (H_2_AETH)BiI_5_, viewed down the *a*‐axis. Dashed lines depict the unit cell outline. Reproduced with permission.^[^ ^45^ ^]^ Copyright 2001, American Chemical Society. l) BX_6_ grouping with “*c*” type stacking of layers where the octahedra are connected by vertices. The A_3_B_2_X_9_ crystal structure type depicted by the atom arrangement in a plane ┴ to the AX _3_ layers. m) 2D corrugated crystal structure (A_3_B_2_X_9_) presented by the atomic arrangement in a plane ┴ to AX_3_ layers. Atoms are represented by large filled circles, B atoms by small open circles and X atoms by large open circles. Reproduced with permission.^[^ ^49^ ^]^ Copyright 1978, International Union of Crystallography. n) Projection view of the 2D corrugated structure (Rb_3_Bi_2_Br_9_). o) Represents that Rb_1_ atoms are coordinated by the characteristic rings of Bi_1_Br_6_ and Bi_2_Br_6_ octahedra. Reproduced with permission.^[^ ^50^ ^]^ Copyright 2016, Wiley‐VCH. p,q) The crystal structures of the Bi‐based double perovskites along the different axis where (r) represents the crystal structure of the typical ABX_3_ perovskites (for comparison purpose).

A family of 0D Bi‐based PVKs exhibits a unique structure (i.e., hexagonal closed packed) amongst all the PVK crystals, which does not share its network in any direction; therefore, known as “0D.” To get a clear idea of the 0D Bi‐based PVKs structure, it is needed to understand the formation of bioctahedra in the structure. Basically, a single octahedron (BiX_6_) is composed of only one Bi atom that resides at the center of the octahedron surrounded by six halogen atoms (X), as shown in **Figure** **4**a. This octahedron is attached to another octahedron via three of its halogen atoms forming a bioctahedra (Bi_2_X_9_), as shown in Figure 4b. In this configuration, Bi‐metal cations cover‐up two‐third of newly formed octahedra sites while one‐third of the metal site remains vacant. The halogens, which are shared by the two Bi atoms (to form bioctahedra) are known as “bridging halides,” whereas the rest of the halides participating in the bonding are known as “terminal halides,” as encircled in Figure 4b. The two bioctahedra are separated by A‐atoms (e.g., Cs, MA, or FA), which resides around the terminal halides; hence, preventing the network to connect further. Together, all these species form a closed packed hexagonal structure,^[^ ^40^, ^41^ ^]^ as depicted in Figure 4c. The hexagonal structure is formed by the combination of a single cation and three halides (i.e., AX_3_), which further combine and form hexagonal stacked layers, i.e., AX_12_ with a common A‐atom, as visualized in Figure 4d. In this way, face shared binuclear octahedra are obtained, which form [Bi_2_X_9_]^3^ **^−^** complex where A‐cations fill‐up the voids at the terminal sides and block any further attachment.^[^ ^42^, ^43^ ^]^ The general formula of 0D Bi‐based PVKs is A_3_Bi_2_X_9_ where MA_3_Bi_2_I_9_ (MBI) with hexagonal structure (space group P_63_/mmc) is extensively studied for various optoelectronic applications.

### 3.2 1D Bi‐Based PVKs

In such Bi‐based PVKs configurations, bismuth halide octahedra (BiX_6_) are attached in such a way that it conforms into a 1D structure. Based on the octahedral connection, 1D Bi‐based PVKs can be subdivided into two categories: i) edge‐shared octahedra and ii) corner‐shared octahedra (zigzag symmetry),^[^ ^12b^ ^]^ which are explained below.

#### 3.2.1 Edge‐Shared Octahedral Structure

To understand the edge‐shared octahedral 1D configuration of Bi‐based PVK, it is necessary to focus on the edge of octahedron that is actually a Bi‐trihalide (BiX_3_), where Bi is residing at the center of three halogen atoms, as marked in Figure 4g. 1D [BiX_4_]^−^ chains are formed when one octahedron (i.e., BiX_6_) shares its opposite edges (i.e., Bi‐trihalide) in such a way that four halogen atoms are bridged together (bridging halides), as shown in the Figure 4e–g. This anionic configuration is repeated throughout in the form of a chain with two free halogen atoms (i.e., terminal halides). The distance of bridging halides and Bi is significantly longer than Bi and terminal halides, which confirms the sharing of edges (i.e., Bi‐trihalides). Subsequently, the distance between Bi atoms of adjoining octahedra is >4.4 Å, which is in agreement with the absence of Bi—Bi bonds; hence, eliminating the chance of the formation of Bi‐octahedra and abstains further attachment.^[^ ^44^ ^]^ Herein, a compound LiBiI_4_⋅5H_2_O is taken as an example of the edge‐shared 1D structure having space group *C*_2_/*c* and monoclinic crystal structure, as shown in Figure 4e–g.

#### 3.2.2 Corner‐Shared Octahedral Structure or Zigzag Chained Structure

In such configuration, the distorted octahedra (BiX_6_) form a 1D network by means of corner‐sharing (i.e., a corner of one octahedron attaches itself with the corner of another via *cis* halogen bridges), hence conforms into a zigzag chain, as shown in Figure 4h. The empty space between these chains is filled by A‐site cations that refrain their mutual interaction; hence, remain isolated.^[^ ^45^, ^46^ ^]^ Therefore, the A‐site cations dictate how the chain should be formed and gives a 1D character to it. The BiX_6_ octahedra build up a zigzag symmetry by means of three different bonding pairs: i) bridging, ii) terminal trans to bridging, and iii) terminal *cis* to bridging. Ideally, bond lengths should be equal but here the case is different, as the bond lengths are such that bridging > *cis* > *trans* bridging bond. The bridging bond, i.e., Bi− I_1_ is the longest amongst the bonds and acts as a backbone of the inorganic chain, while Bi− I_1_, Bi− I_2_, and Bi− I_3_, Bi− I_4_ are cis and trans, respectively to the long bridging bonds,^[^ ^47^ ^]^ as shown in Figure 4h,i. The example of the 1D zigzag structure is (H_2_AETH)BiI_5_ PVK with an orthorhombic crystal system, as indicated in Figure 4j,k.

### 3.3 2D Bi‐Based PVKs

A typical 0D (A_3_Bi_2_I_9_) Bi‐based PVK structure converts into a 2D corrugated layered defect PVK structure in two ways: a) replacing A‐site cation (e.g., K^+^, Rb^+^, and NH_4_^+^) with reduced size and b) replacing halogen atoms(e.g., Br and Cl).^[^ ^48^ ^]^ Consider the same 0D Bi‐based PVK structure (discussed above) where AX_3_ layers formed a hexagonal closed pack structure (Figure 4d). These AX_3_ layers can have different modes of stacking namely; i) (h)_6_, ii) (hcc)_2_, and iii) (c)_3_, where one can find the separation of double octahedra Bi_2_X_9_ caused by (h)_6_ stacking mode.^[^ ^49^ ^]^ Bonding of a single halogen atom with octahedra is possible due to (c)_3_ stacking mode whereas (hcc)_2_ stacking mode allows both of the above networking.

2D Bi‐based PVKs crystal structure is closed to cubic (c)_3_ packing type, where AX_3_ layers are stacked as a cubic layer and form more complicated polyhedral symmetry, as shown in Figure 4l. These layers are distorted and unlike the 0D structure, one cannot find AX_12_ layers. It is because of the enormous difference in the ionic radii of the A‐site cation and X‐site halogen atoms, where no close packing is possible.^[^ ^41^ ^]^ Moreover, BiX_6_ octahedra are also distorted and build up a structure in which three of X atoms are nearer (termed as bridging halides) while the other three atoms are farther from Bi, Figure 4b. These bridging halides along with Bi (i.e., BiX_3_) set inside AX_3_ layers in such a way that all bridging atoms are shared with three other octahedra, forming corrugated Bi_2_X_9_ layers, as shown in Figure 4m. These corrugated layers run throughout such that it forms a 2D structure,^[^ ^48^, ^50^, ^51^ ^]^ as shown in Figure 4n,o. Herein, Rb_3_Bi_2_I_9_ PVK compound with monoclinic structure (space group *Pc*) is taken as an example of a 2D corrugated structure, see Figure 4n.

### 3.4 3D Bi‐Based PVKs

3D Bi‐based PVKs are also known as double perovskites or elpasolites, having a general formula A_2_B′B″X_6_, where A = cation, B′ = monovalent cation, B″ = trivalent cation, and X = halogens. To achieve such a 3D‐PVK structure, a heterovalent substitution of Pb^2+^ is required. This can be done by replacing a B‐site cation into two different elements such that one half of B‐site cation acquire a charge of 3+ and the other half acquire 1+ charge to preserve charge neutrality. Due to the replacement of a single metal cation in the B‐site, the overall crystal lattice expands twice as compared to the conventional 3D ABX_3_ PVK structure. Therefore, the structure is named as “double perovskite.”^[^ ^12^, ^52^ ^]^ Moreover, in the case of conventional 3D ABX_3_ PVKs, a B‐site cation shares 6‐fold coordination, while in the case of double PVKs, a similar 3D structure is composed of alternative corner‐shared octahedra of B′X_6_ and B″X_6_ with cuboctahedral voids filled by A‐cations,^[^ ^53^ ^]^ as depicted in Figure 4p–r. Herein, we considered the most commonly studied double PVK compound, i.e., Cs_2_AgBiBr_6_ (space group Fm_3_m) as an example of elpasolite, see Figure 4p,q and its comparison with conventional CsPbBr_3_ PVK Figure 4r.

## 4 Optoelectronic Properties of Bi‐Based PVKs

Better optoelectronic properties are prerequisites for optoelectronic devices, which make them the ultimate choice for better performance. These properties include photoabsorption, energy bandgap and its nature, photoemission, charge carrier mobilities, photoresponsivity, defect concentration, exciton binding energy, charge carrier lifetime, etc. In the following section, the important optoelectronic properties have been discussed with reference to bismuth.

### 4.1 Optical Absorption

**Figure 5:**
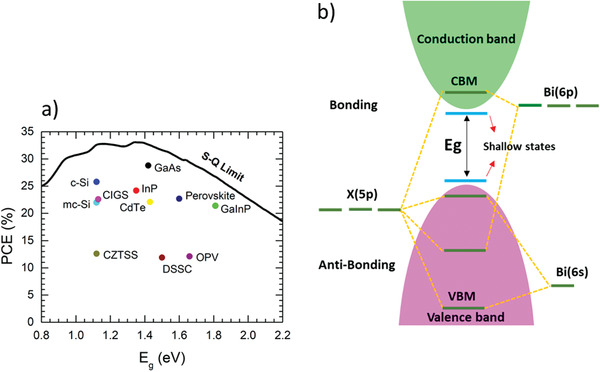
a) Maximum PCE (Shockley–Queisser limit) for a solar‐cell operated under AM 1.5G illumination at 298.15 K, as a function of the energy bandgap. Reproduced with permission.^[^ ^54^ ^]^ Copyright 2018, American Chemical Society. b) Electronic band structure of the Bi‐based perovskites and formation of shallow states, indicating their defect tolerance nature.

On average, the earth receives about 71% of the total solar spectrum where the rest of 29% reflects back to the atmosphere. For harnessing solar energy efficiently, a single junction device is needed to have a bandgap (≈1.2‐1.3 eV) to fulfill the Shockley–Queisser limit,^[^ ^21^, ^54^ ^]^ **Figure** **5**a. Conventional Pb‐based ABX_3_PVKs have energy bandgap near to this limit but as far as Bi‐based PVKs are concerned, they have relatively larger energy bandgaps. Nevertheless, the preliminary study reveals that MBI and Cs_3_Bi_2_I_9_ (CBI) exhibit comparable optical absorption to the conventional Pb‐based PVKs.^[^ ^55^ ^]^

The classification of Bi‐based PVKs (i.e., 0D, 1D, 2D, and 3D) distinguishes them from conventional ABX_3_ type PVKs. In Bi‐based PVKs, [BiX_6 _]^‐^octahedral units constitute valence and conduction electronic states; therefore, structural dimensionality has a strong impact on optoelectronic properties.^[^ ^56^ ^]^ A detailed explanation of the optoelectronic properties of the overall Bi‐based PVKs is beyond the scope of this report. Herein, our discussion will be limited to the most frequently studied Bi‐based 0D PVK, i.e., A_3_Bi_2_X_9_ with A = Cs, FA, and MA. However, the optoelectronic properties of some other important Bi‐based PVKs will also be summarized.

#### 4.1.1 Optical Bandgap

**Figure 6:**
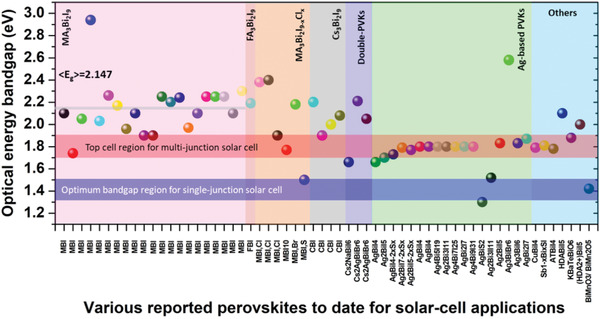
Summary of the overall reported optical bandgaps of Bi‐based perovskites utilized for solar cells. The horizontal gray line in the pink shaded portion represents the average bandgap line of the most extensively studied perovskite compound, i.e., MA_3_Bi_2_I_9_ for solar‐cell applications. The blue and red horizontal shaded portions indicate the optimum bandgap regions of the single and multijunction (top cell) solar cells. Most of the reported bandgaps are in the optimal bandgap region of the multijunction solar cell (top cell). The data was collected from the Web of Science by searching the keywords “Bismuth + perovskites + solar cells.”

Bi‐based PVKs share the same electronic configuration as that of lead‐based PVKs, i.e., their conduction band electronic states comprise of partially filled Bi‐p orbitals and fully occupied valence band with Bi‐s and halides‐p orbitals. The energy bandgap is established between conduction band minimum and valence band maximum, as displayed in Figure 5b. However, due to the existence of an intense excitonic peak near 500 nm, it is difficult to calculate the true bandgap for the lower‐dimensional material including 0D Bi‐based PVKs.^[^ ^57^ ^]^ Unlike the Pb‐based conventional 3D‐PVKs (where the excitonic peak is predominated by the absorption band‐edge and the true bandgap could precisely be estimated); the case is twisted for lower dimensional Bi‐based PVKs, i.e., a strong excitonic peak is tempered with the band edge, masking the true bandgap. Therefore, a discrepancy is always found in the reported bandgaps of the Bi‐based PVKs. For example, the reported bandgaps for CBI are ranged from 1.9 to 2.8 eV.^[^ ^41^ ^]^ Similarly, the energy bandgap of the MBI is within the range of 1.75–2.94 eV. The bandgaps of the overall Bi‐based PVKs reported to date for solar‐cell applications are summarized in Figure 6. The average bandgap of the MBI (calculated from the reported bandgaps) is found to be 2.147 eV. The bandgaps of the phase pure Bi‐based PVKs are much higher than the optimal bandgap of the single‐junction solar‐cell. However, they mostly exhibit a bandgap in the optimal region of the top cell in the multijunction tandem solar‐cells, as highlighted in **Figure** **6**.

**Figure 7:**
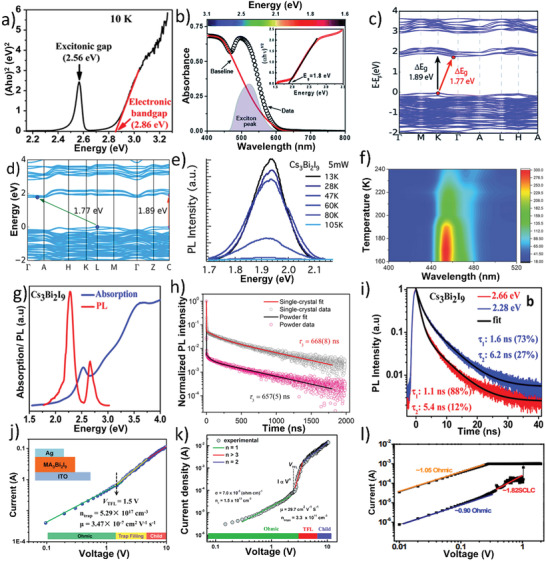
Optoelectronic properties of various Bi‐based perovskites: a) Tauc plot of Cs_3_Bi_2_I_9_ nanocrystals at 10 K. Reproduced with permission.^[^ ^57^ ^]^ Copyright 2018, American Chemical Society. b) Absorption spectra of the MBI film. After the exciton peak was extracted, an indirect bandgap of 1.8 eV was calculated. c) Band structure of MBI calculated by DFT: 1.77 eV (indirect bandgap, red arrow) and 1.89 eV (direct bandgap, black arrow). Reproduced with permission.^[^ ^63^ ^]^ Copyright 2017, The Royal Society of Chemistry. d) Electronic band structure of CBI (based on PBE+SOC) along with high symmetry points in the reciprocal space, exhibiting an indirect bandgap (1.77 eV) b/w L‐point in the valence band and along Γ → A direction in the conduction band. The direct bandgap of 1.89 eV has occurred at the C‐point. Reproduced with permission.^[^ ^61^ ^]^ Copyright 2016, American Chemical Society. e) PL spectra of CBI measured as a function of temperature. Reproduced with permission.^[^ ^64^ ^]^ Copyright 2017, American Chemical Society. f) Pseudocolor map of the temperature‐dependent PL spectra of CBI single‐crystal. Reproduced with permission.^[^ ^73^ ^]^ Copyrights 2017, Wiley‐VCH. g) Optical absorption and PL spectra of the CBI NCs. Reproduced with permission.^[^ ^57^ ^]^ Copyright 2018, American Chemical Society. h) Time‐resolved room‐temperature PL decay profiles of the powder and single‐crystal samples. Reproduced with permission.^[^ ^85^ ^]^ Copyright 2016, American Chemical Society. i) PL decay profiles of colloidal CBI NCs measure at two different emission wavelengths (i.e., 466 and 544 nm). Reproduced with permission.^[^ ^57^ ^]^ Copyright 2018, American Chemical Society. j,k) Are SCLC curves of the ITO/MBI/Ag and ITO/MBI/Au (electron only) devices plotted under double‐logarithmic scales measured in positive sweep modes. Reproduced with permission.^[^ ^92^, ^152^, ^153^ ^]^ Copyright 2017, American Chemical Society, 2016, The Royal Society of Chemistry, and 2018, The Royal Society of Chemistry.

The occurrence of the excitonic peak for 0D PVK (i.e., CBI) nanocrystals was reported at 2.56 eV where the bandgap (determined from the absorption onset) was observed at 2.86 eV,^[^ ^57^ ^]^ see **Figure** **7**a. The existence of these excitonic peaks was further confirmed from the absorption spectra measured at different temperatures (296–10 K). The same results were obtained for the nanocrystals as well as for the bulk CBI. Additionally, it was revealed that these excitonic peaks are not due to the structural size but are the features of dimer like structure.^[^ ^58^ ^]^

Furthermore, it has been reported that single crystal and thin films of the same Bi‐based PVK compound exhibited different optical bandgaps. For instance, dissimilar bandgaps for the single crystal (1.96 eV), polycrystalline powder (2.0 eV), and thin films (2.26 eV) were determined.^[^ ^59^ ^]^ It was suggested that the possible reasons for this variation in the bandgaps could be; i) crystal orientation, ii) crystallinity of material, and iii) defects concentration in the material. Usually, a single crystal has lesser defect sites with higher crystallinity, which results in a smaller bandgap. This is further justified when the single crystal is ground and observed, the deep red color is changed to an orange blue color, which indicates a blue shift in the wavelength. Moreover, this effect became even more pronounced in the case of thin films, where the defect sites and orientation are affected more.^[^ ^59^ ^]^

#### 4.1.2 Nature of the Bandgap

Besides the width of the optical bandgap of a photoabsorber, the nature of the bandgap is also important. In the case of direct bandgap material, most of the absorbed light is converted into radiative transitions. Conversely, in the case of the indirect bandgap material, majority of the absorbed radiations are emitted as nonradiative transitions (e.g., thermal phonons, etc.); hence, most of the energy is lost.^[^ ^60^ ^]^ As far as the energy bandgap of the 0D Bi‐based PVKs is concerned, MBI possesses a direct bandgap while both direct (at higher energy) and indirect (at lower energy) bandgaps are reported for the CBI.^[^ ^60^, ^61^, ^62^ ^]^ However, in some cases, an indirect bandgap for MBI is also reported, see Figure 7b–d.^[^ ^62^, ^63^ ^]^ It is noteworthy that in contrast to the 0D Bi‐based PVKs (i.e., MBI and CBI), the 2D Bi‐based PVKs (Rb_3_Bi_2_I_9_) nanocrystals (NCs) exhibited two prominent sharp absorption peaks at 2.28 eV (546 nm) and 2.65 eV (468 nm), respectively.^[^ ^57^ ^]^ Similar absorption characteristics have also been reported in bulk Rb_3_Bi_2_I_9_ and other 2D perovskites like Cs_3_Bi_2_Br_9_ (CBBr) and hybrid Ruddlesden–Popper perovskite.^[^ ^64^, ^65^ ^]^ But neither Rb**_3_**Bi**_2_**I**_9_** nor NCs have shown any measurable PL, which might have specified the presence of native defect states or nonradiative recombination in the bandgap region.^[^ ^64^ ^]^

#### 4.1.3 Absorption Coefficient

The optical absorption coefficient of a material is crucial to exploit it for optoelectronic applications. In this regard, Bi‐based PVKs showed ideal photoabsorption characteristics with an optical absorption coefficient as high as 10^5^ cm^−1^.^[^ ^11^, ^55^ ^]^ Bi‐based PVKs performed well in both visible and infrared regions. The absorption between X‐5p valence states and Bi‐6p absorption states are responsible for these excellent absorption properties of Bi‐based PVKs. Moreover, its better absorption can also be attributed to the optical transition from Bi‐6s states (near the valence band maximum) to Bi‐6p conduction states.^[^ ^66^ ^]^

### 4.2 Photoluminescence

It is a spontaneous emission of light from a material (when optically excited), which helps to provide a direct analysis of the optical properties of surfaces and interfaces.^[^ ^67^ ^]^ In the case of defect‐free material, photoluminescence (PL) peaks and absorption band edge are expected to occur at the same energy but for materials having defects, PL peak may deviate from the absorption band edge, known as Stoke's shift, which could be intrinsic or extrinsic in nature.^[^ ^68^ ^]^ Moreover, defects in the structure cause a broadening of the PL peak, which is common in Bi‐based PVKs.^[^ ^69^ ^]^ BiX_6_ octahedra in Bi‐based PVKs are prone to structural deformation as they need to maintain charge neutrality.^[^ ^70^ ^]^ Such structural deformation generates polarons, which are analogous to free electrons and create optically active states between valence and conduction bands, known as “self‐trapping states”; hence forms emission centers.^[^ ^70^, ^71^ ^]^ In this way, excited electrons experience nonradiative transitions before reaching the respective energy levels. The occurrence of these self‐trapping states produces an emission with lower energies, which is the reason for red‐shifted wavelength corresponding to a large Stoke's shift and is intrinsic in nature. The occurrence of these trapping states is also observed theoretically by Mosconi et al.^[^ ^72^ ^]^

Furthermore, the PL of A_3_Bi_2_X_9_ single crystals has been experimentally measured at room temperature as well as at lower temperatures,^[^ ^64^ ^]^ as shown in Figure 7e,f. For CBI, a less intense and broad emission peak (ranging from 1.85 to 2.03 eV and centered at 1.93 eV) was observed; whereas, Rb_3_Bi_2_I_9_ did not show any PL response at room temperature. The broadening in the PL spectrum of the CBI was attributed to the local lattice distortion or octahedral distortion.^[^ ^64^ ^]^ Additionally, it was also observed that the PL properties of CBBr were sensitive to the phase transition, caused by a decrease in temperature; therefore, the mechanism of PL of CBBr was studied in the temperature range of 160–240 K.^[^ ^73^, ^74^ ^]^ A red‐shift from 453 to 457 nm along with a decrease in PL intensity was observed and attributed to the nonradiative recombination initiated by the temperature above 200 K. In addition to single crystals, the PL properties of thin films and quantum dots (QDs) were also studied. For instance, all inorganic Cs_3_Bi_2_X_9_ (X = Cl, Br, and/or I) QDs have demonstrated even a high PL quantum yield than the Pb‐based inorganic analogs. Along with excellent PL properties, such QDs exhibited extraordinary moisture and photostability, which were attributed to the negation of the defects caused by the smaller size effect.^[^ ^75^ ^]^

Other than the typical single broad PL peak, two separated PL emission peaks (at 2.66 and 2.28 eV) with narrow full‐width‐at‐half‐maxima were also reported for CBI NCs,^[^ ^57^ ^]^ see Figure 7g. To determine the origin of these peaks, PL spectra of various crystals (with different sizes and shapes) were studied. It was concluded that these emission peaks did not result from the different sizes and shapes of the crystals. Indeed, the peak at 2.6 eV was attributed to the deexcitation emission while the peak at 2.28 eV was attributed to the excitonic emission.

### 4.3 Electronic Band Structure and Defect Tolerance

Despite the existence of defects, the capability of semiconductor to maintain its optoelectronic properties is termed as defect tolerance nature of the material. Defects are inevitable during material processing (especially during solution processability). Optoelectronic properties are not severely affected by the defects formed exterior to the bandgap. Conversely, intraband deep level defects have decisive effects on the optoelectronic properties of a material. These defects subsequently pave a way for the nonradiative recombination or become trap centers by itself, hence restricting the charge transport.^[^ ^76^ ^]^

Antibonding interaction at valence band maximum causes the defects to confine to shallow states at the band edges. Bi‐based PVKs are presumed to have defect tolerance capability due to active ns^2^ lone pairs, which tend to create antibonding interactions.^[^ ^77^ ^]^ To understand this, consider the electronic band structure of Bi‐halide, where Bi‐6p orbital hybridizes with halogen X‐5p orbitals, forming deeper states of the valence band as well the bottom part of the conduction band (conduction band minima). Similarly, Bi‐6s and X‐5p overlap to form the valence band minima (bonding) and valence band maxima (antibonding). Only one X‐5p orbital interacts with Bi‐6s and Bi‐6p orbitals, which form the middle of the valence band.^[^ ^78^ ^]^ Consequently, these interactions create the energy bandgap between valence band maxima (VBM) and conduction band minima (CBM), as presented in Figure 5b. The formation of this electronic structure compels the defects to get closer to the band edge. In other words, shallow defect states are formed between VBM and CBM, which allow easier charge carriers transport and hence increase their mobility.^[^ ^20^, ^77^, ^79^ ^]^

Additionally, it has been reported that the nature of VBM formation of CBI is the same as in MAPbI_3_ while the nature of the CBM is different from Pb analog.^[^ ^80^ ^]^ It was also observed theoretically that MBI was expected to have a better defect tolerance nature in comparison to CBI.^[^ ^80^ ^]^ Likewise, it was reported previously for Bi‐based double PVKs (Cs_2_AgBiBr_6_) that due to the weak coupling of Bi‐6s and Br‐6p, it contributes less to the VBM. As a result, Ag acted as a shallow acceptor vacancy and could form p‐type conduction; hence, did not show better defect tolerance.^[^ ^81^ ^]^ Furthermore, it was suggested that the synthesis of Bi‐based double PVKs need to fulfill Br‐poor or Bi‐rich growth conditions to make these materials defects tolerant.

### 4.4 Carrier Lifetime

Longer carrier lifetime is essential for better charge extraction that consequently leads toward better photovoltaic performance.^[^ ^82^ ^]^ A short laser pulse induces excitation in the material, as a result, exciton (electron–hole pair) is created that upon Coulombic‐interaction wants to recombine with each other. Time‐resolved PL measures the time of the excited electron until it recombines, known as carrier lifetime. For longer, the charge carriers (electron–hole pair) are separated, longer carrier lifetime is expected. Longer carrier lifetime suggests that charge extraction and transport becomes more efficient, which makes the material more favorable for the photovoltaic applications.^[^ ^83^ ^]^

Reported lifetime for Pb‐free substitutes ranged between <0.1 ns to ≈10 ns.^[^ ^84^ ^]^ However, the case of Cs_2_AgBiBr_6_ and Cs_2_AgBiCl_6_ was found to be an exception, i.e., they exhibited a faster exponential decay followed by a slower decay tail.^[^ ^85^, ^86^ ^]^ Therefore, to fit those time‐resolved PL (TRPL) curves, bi‐ or even triexponential functions were used. The initial faster decay was attributed to the recombination centers or crystallographic defects, whereas the longest decay tail was accredited to the fundamental exciton lifetime, see Figure 7h,i.^[^ ^87^ ^]^ The possible reason for this longer carrier lifetime could be the indirect bandgap and defect tolerance nature of the material.^[^ ^81^, ^84^ ^]^ Moreover, it was reported that the decay lifetime was significantly different for single‐crystal (145 ns) and powder (54 ns) of Cs_2_AgBiBr_6_, which suggested that a powder sample has more defect sites. However, longer PL decay lifetime reported (i.e., 668 and 657 ns), which were not significantly different for the two samples and attributed to the fundamental recombination lifetime, as shown in Figure 7h.^[^ ^85^ ^]^

### 4.5 Exciton Binding Energy

Exciton binding energy is another essential parameter that governs the optoelectronic properties of the devices. Excitons having smaller binding energy can be separated easily, which prolongs their lifetime and carrier diffusion length. Due to their lower dimensionality and structure isolation, OD‐PVK structure (e.g., CBI) resulted in a higher exciton binding energy, exceeding a few 100s meV. However, they have the potential to exhibit optical properties comparable to the Pb‐based 3D metal halide PVKs.^[^ ^64^ ^]^ For example, Machulin et al.^[^ ^58^ ^]^ reported a higher exciton binding energy of 279 meV for CBI 0D‐PVK. It was suggested that such a higher exciton binding energy could be the reason behind the ambiguity of the bandgap and crystal color. Similar higher exciton binding energy was reported in the ionic alkali halide compounds, which caused a larger Stoke's shift in absorption and emission spectra.^[^ ^88^ ^]^ Such high binding energy was responsible for the excitonic absorption peaks even at room temperature.^[^ ^89^ ^]^ Similarly, even higher exciton binding energy of 300 meV was reported for the MBI 0D‐PVK.^[^ ^64^ ^]^

### 4.6 Charge Carrier Mobility

Charge carrier's mobility is drift velocity per unit electric field, which defines how fast the charge carriers move at a given field strength. In other words, it means how swiftly the charge carriers move through metals or semiconductors when dragged by the applied electric field. Based on films without and with electrodes, there are many ways to estimate the charge carrier mobilities.^[^ ^90^ ^]^ Herein, our discussion will be limited to the space‐charge‐limited‐current (SCLC) method, which is frequently used for the calculation of the charge carrier mobility of the Bi‐based PVKs. In SCLS, current–voltage (*IV*) characteristics follow Lampert's theory of current in solids, which is divided into three regions, as indicated in Figure 7j,k.^[^ ^59^ ^]^ i) At a lower voltage, the region (known as linear or Ohmic region), follows *I* ∝ *V*. ii) At higher biasing conductions carried out by traps, follows *I* ∝ *V^n^* ^> 3^, known as trap‐filled limit (TFL) region. This region is helpful to estimate the trap concentration in the material. iii) The next is a Child regime, which is followed by Mott Gurney relation, i.e., *I* ∝ *V^n^* ^= 2^, where mobility can be estimated by the Equation (1)

(1) $$\mu =8JL^{3}/9\varepsilon \varepsilon_{0}V^{2}$$

where *μ* is the mobility, *J* is the current density, *L* is the thickness of the material (thin film), *ε* is the dielectric constant of the material, *ε*_0_ is the permittivity of free space, and *V* is the applied voltage. Using SCLS calculation, the mobility of MBI was reported to be 29.7 cm^2^ V^−1^ s^−1^, which was comparable to the lead analogs, showed its applicability for the photovoltaics, see Figure 7j–l.^[^ ^59^, ^91^ ^]^ To ensure better carrier mobility, the quality of the thin film is essential, because a rough film (having pinholes) could lower the carrier mobility. For example, significantly lower carrier mobility, i.e., 3.47 × 10^−7^ cm^2^ V^−1^ s^−1^ was reported for the similar MBI PVK thin film, which was attributed to the inferior quality of the film, see Figure 7j.^[^ ^92^ ^]^

## 5 Optoelectronic Applications of Bi‐Based PVKs

**Figure 8:**
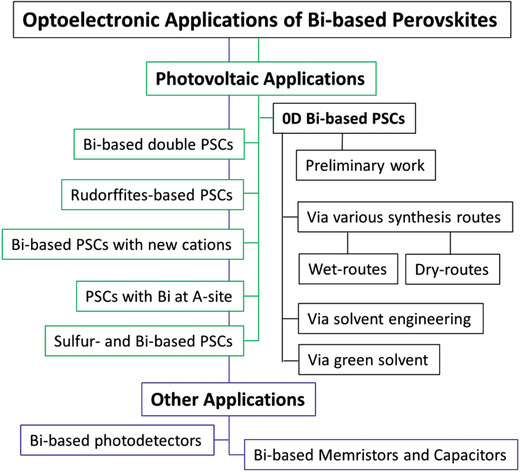
A flowchart of the optoelectronic applications based on Bi‐based PVKs.

Due to their exceptional optoelectronic properties and excellent stability at standard operational conditions, Bi‐based PVKs have been used extensively for several optoelectronic applications including photovoltaics, photodetectors, memory devices, and capacitors. It provided a diverse structural platform for the novel nontoxic PVKs, where different A, B, and X‐site cations and anions have been exercised for numerous applications. Being A‐site organic cations, MA and Cs remained an essential part of the state‐of‐the‐art Pb‐based PVKs solar‐cells; therefore, as compared to the other A‐site cations, most of the Bi‐based PVKs are accompanied with MA and Cs as A‐site cations. Thus, in the first part of the succeeding section, some of the preliminary works based on the 0D Bi‐based PSCs (with Cs and MA as A‐site cations) will be conferred. Since high quality and compact Bi‐based PVK thin films remained a challenging issue for all the time; therefore, the ongoing discussion will be subdivided on the basis of numerous synthesis routes as well as solvent‐engineering and green solvents. In the next section, PSCs based on other types of Bi‐based PVKs will also be elaborated in detail. At the end of the section, a summary of the overall progress made on Bi‐based PSCs will be compiled. Moreover, optoelectronic applications other than PSCs will also be elucidated in detail, see a flowchart, **Figure** **8**.

### 5.1 0D MBI and CBI‐Based PSCs

Undeniably, the hallmarked lead‐based PVKs are renowned for their unprecedently increased performance in solar‐cells.^[^ ^93^ ^]^ Owing to their comparable optoelectronic properties with Pb‐based PVKs, it is expected that Bi‐based PVKs could also provide the same solar‐cell characteristics. Therefore, followed by the pioneering effort of Park et al.^[^ ^55^ ^]^ in 2015, Bi‐based PVKs are rigorously exploited for photovoltaic applications.^[^ ^78^, ^94^ ^]^

**Figure 9:**
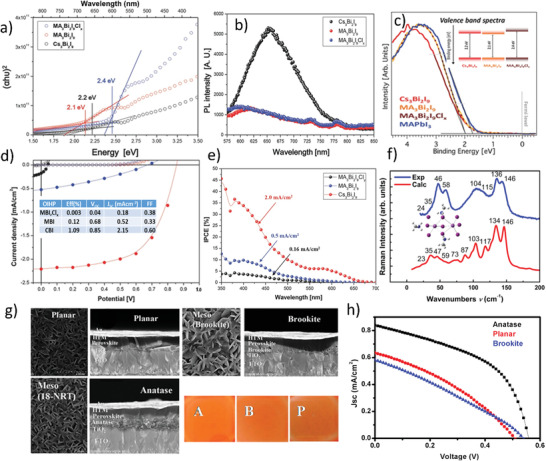
a,b) Tauc plots for energy bandgaps calculations and Photoluminescence spectra. c) Valance band spectra of Bi‐based PVKs and in the inset is their comparison with the typical lead‐based PVK MAPbI_3_. d) *JV*‐characteristics, where, the inset of the figure is device parametric metrics. e) The IPCE curve for the three different Bi‐based PVKs. Reproduced with permission.^[^ ^55^ ^]^ Copyright 2015, Wiley‐VCH. f) Raman spectra of MBI (both experimental and theoretical). Reproduced with permission.^[^ ^98^ ^]^ Copyright 2016, Elsevier. g) SEM images of MBI PVKs (top and cross‐section) layers deposited on c‐TiO_2_, brookite m‐TiO_2_, and anatase m‐TiO_2_ the inset of the figure is the appearance of the films A = planar, B = brookite, and C = anatase. h) *JV*‐characteristics of the best performance MBI‐based solar‐cell device prepared on the planar, brookite, and anatase substrates. Reproduced with permission.^[^ ^99^ ^]^ Copyright 2016, American Chemical Society.

In the preliminary work of Park et al.,^[^ ^55^ ^]^ three types of Bi‐based PVKs thin films, i.e., CBI, MBI, and MA_3_Bi_2_I_9_Cl*_x_* have been synthesized via simple solution processability and spin‐coating. All these PVKs were prepared under ambient environmental conditions and their optical properties were studied that indicated long‐term stability. It was realized that like Pb‐based PVKs, the optical properties of Bi‐based PVKs also varied with the variation of the cations (MA or Cs), see **Figure** **9**a–c. The absorption coefficients of all these compounds were determined to be ≈2 × 10^5^ cm^−1^ and their bandgaps and exciton binding energies were found to be in the range of 2.1–2.4 eV and 70–300 meV, respectively. Although their absorption coefficients were comparable to the conventional Pb‐based PVKs, their bandgaps and exciton binding energies were found to be way larger than Pb‐based PVKs. Finally, solar‐cells based on the above three PVK compounds were fabricated where the highest PCE of 1.09% was demonstrated by the CBI‐based device, see Figure 9d,e. Very similar work was presented by Lyu et al.^[^ ^95^ ^]^ where a first single‐crystal and solution‐processed thin films of MBI were demonstrated for photovoltaic applications. The solar‐cell device exhibited a slightly higher PCE of 0.192% with open‐circuit voltage (*V*_OC_) of 0.51 V, short‐current density (*J*_SC_) of 1.16 mA cm^−2^, and fill factor (FF) of 46%. Likewise, Zhijie et al.^[^ ^96^ ^]^ synthesized a millimeter‐sized single crystal of A_3_Bi_2_I_9_ (A = methylammonium or MA and Cs) via a hydrothermal process. The prepared crystals exhibited a bandgap of 1.9 eV and spin‐coated thin films showed a uniform surface morphology, lower trap densities, significantly higher carrier mobility, and extended environmental stability. Finally, a solar‐cell device based on MBI PVK yielded a PCE of ≈0.2% with *V*_OC_ 0.53 V, *J*_SC_ 0.65 mA cm^−2^, and FF of 65%. Similarly, an air‐stable single crystal, powder, and thin films of MBI were synthesized and their optoelectronic properties were observed to be sensitive to the crystallinity of the compound.^[^ ^97^ ^]^ For instance, different bandgaps for a single crystal (1.96 eV) and thin film (2.26 eV) were observed. Additionally, an outstanding photocurrent generation in the visible range and longer exciton lifetime along with higher mobilities were revealed by transient absorption spectroscopy (TAS) and SCLC measurements, respectively. Unfortunately, a solar‐cell device underperformed with PCE of 0.11% along with *V*_OC_ 0.72 V, *J*_SC_ 0.49 mA cm^−2^, and FF of 31.8%, which was attributed to the poor film morphology. Subsequently, Oz et al.^[^ ^98^ ^]^ reported the preparation and structural properties of 0D MBI. The prepared PVK compound showed a relatively wider bandgap of 2.9 eV with a PL peak at 1.65 eV. The vibration modes of the bioctahedra [Bi_2_I_9_]^3−^ were confirmed by their lower wavelength (>200 nm) Raman peaks, see Figure 9f. A heterojunction solar‐cell of this material yielded a PCE of 0.1%, with *V*_OC_ of 0.66 V, *J*_SC_ of 0.22 mA cm^−2^, and FF of 0.49%.

In the aforementioned initial works, the PCE of MBI based solar‐cells was substantially lower (i.e., 0.12% and 0.1% respectively) as compared to the CBI (with slightly higher PCE). There could be many reasons for the lower PCE including; poor film morphology and crystallinity, wider bandgap and deeper defect states, greater exciton binding energy, and material surrounding the active PVK layer, etc. Therefore, the effect of the electron transporting layer on the performance of MBI‐based solar‐cells was studied.^[^ ^99^ ^]^ A polycrystalline film was fabricated by a one‐step solution process and three types of device architectures, i.e., planar, on brookite meso‐TiO_2_, and anatase meso‐TiO_2_ were investigated. It was comprehended that the film morphology was significantly reliant on the choice of the substrate and device architecture. For instance, PVK films grew nonuniformly on the planar substrates; however, films grown on the anatase meso‐TiO_2_ layers exhibited better nucleation and uniform film growth, see Figure 9g. Unlike, in the case of brookite‐TiO_2_, the interparticle‐necking impeded the MBI percolation in the pores; thus, hindered the nucleation and uniform growth of the PVK film. Finally, solar‐cell devices fabricated on the anatase meso‐TiO_2_ exhibited better performance (e.g., *J*_SC_ 0.8 mA cm^−2^) than one fabricated on the brookite meso‐TiO_2_ layer, see Figure 9h. Solar‐cell devices showed a slightly improved PCE of 0.2% with enhanced stability of more than 10 weeks in the ambient conditions. Similarly, Zhang et al.^[^ ^100^ ^]^ synthesized MBI PVK through a one‐step solution process and systematically investigated the development of the film morphology on the meso‐TiO_2_/ITO surface. It was realized that the concentration of the precursor solution and the structure of the substrate were crucial for a highly crystalline PVK film. Consequently, the PCE of the solar‐cell was successfully improved to 0.42% with *V*_OC_ 0.66 V, *J*_SC_ 0.91 mA cm^−2^, and FF of 65%.

A little modification in the stoichiometry of CBI was made and a new PVK compound, i.e., Cs_3_Bi_2_I_10_ was introduced,^[^ ^101^ ^]^ which exhibited an extended absorption spectrum with a relatively shorter bandgap (1.77 eV). In comparison with CBI, that new compound (i.e., Cs_3_Bi_2_I_10_) exhibited a layered structure with different crystal orientations. Due to its broad OA spectrum, a solar‐cell made of Cs_3_Bi_2_I_10_ PVK showed an extended photocurrent up to 700 nm, which was possibly indicative of its increased optical absorption and photocurrent of the solar‐cell. Device characteristics showed a PCE of 0.4% with *V*_OC_ 0.31 V, *J*_SC_ 3.40 mA cm^−2^, and FF of 38%.

### 5.2 Performance of 0D Bi‐Based PSCs Prepared via Different Fabrication Routes

**Figure 10:**
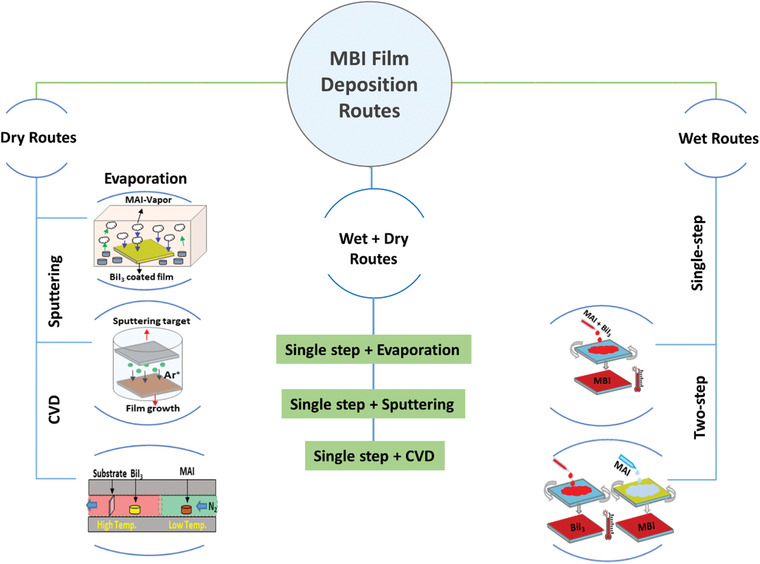
Schematics of the film deposition routes for the fabrication of high quality MBI thin films for improved photovoltaic performance.

The morphology of PVK thin film is crucial for superior PV performance. For instance, better crystallinity, full coverage of the substrate, and the vertically oriented (perpendicular to the substrate) PVK crystals are essential for the minimized shunt resistances, enhanced photoabsorption, and better charge transport. Unlike Pb‐based PVKs, Bi‐based PVKs crystalize directly in a rapid way without forming any intermediate solvated phases, which lead to isolated crystals instead of growing into a uniform pinholes‐free films.^[^ ^102^ ^]^ Thus, it is challenging to fabricate compact thin films of Bi‐based PVKs. Therefore, along with conventional one‐step solution processability, many other techniques have been utilized to fabricate high‐quality Bi‐based PVKs thin films for improved PV device performance. Those techniques included: a) wet chemical approaches, including a single‐step and two‐step solution processability,^[^ ^103^ ^]^ b) dry routes, including vacuum vapor depositions^[^ ^104^ ^]^ or evaporation,^[^ ^105^ ^]^ magnetron sputtering, etc.,^[^ ^106^ ^]^ c) other techniques based on the combination of dry and wet routes and electric field‐assisted spray coating, etc.,^[^ ^107^ ^]^ as summarized in **Figure** **10**. The progress in the PV‐performance of MBI‐based devices (synthesized through these routes) will be conferred in detail in the following sections.

#### 5.2.1 0D Bi‐Based PSCs via Modified One‐Step Solution Process

**Figure 11:**
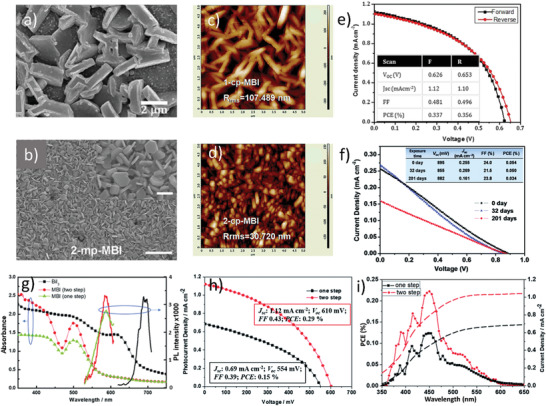
Comparison of the surface morphology and PV‐performance of the single‐step and two‐step processed films: a) SEM image of the MAIB thin film deposited by one‐step anti‐solvent assisted crystallization process. Reproduced with permission.^[^ ^154^ ^]^ Copyright 2017, Wiley‐VCH. b) Top‐view FE‐SEM images of the MBI perovskite layer MBI on m‐TiO_2_ prepared via two‐steps solution process. c,d) AFM images of MBI films deposited on a c‐TiO_2_ substrate via one and two‐step process, respectively. Reproduced with permission.^[^ ^110^ ^]^ Copyright 2017, The Royal Society of Chemistry. e) *J*−*V* curves and hysteresis analysis of the MBI‐based perovskite solar cells in forward and reverse scan direction, prepared via single‐step antisolvent assisted crystallization. The inset is device parameter metrics for reverse and forward scans. Reproduced with permission.^[^ ^154^ ^]^ Copyright 2017, Wiley‐VCH. f) PV parametric matrix and *JV*‐curves along with device stability, prepared via a two‐step solution process. Reproduced with permission.^[^ ^110^ ^]^ Copyright 2017, The Royal Society of Chemistry. g) Comparative PL and optical absorption spectra of BiI_3_ and MBI films prepared via single and two‐step solution processes. h) Device parameters and *I*–*V* performance for the devices fabricated by one and two‐step solution processability measured under 1 sun illuminations and i) Corresponding IPCE results. Reproduced with permission.^[^ ^112^ ^]^ Copyright 2017, The Royal Society of Chemistry.

The conventional one‐step solution process has been commonly adopted to fabricate PVK thin films with the following advantages: less time consumption, facileness, and cost‐effectiveness. However, due to the rapid crystallization of precursors, conventional one‐step solution processability lead toward certain disadvantages; including poor crystallinity, less coverage of the substrate, and the formation of noncompact and disoriented PVK films.^[^ ^55^, ^95^, ^97^, ^98^ ^]^ Therefore, the one‐step route was modified and vertically oriented MBI PVKs films were fabricated via a gas‐assisted one‐step solution process.^[^ ^108^ ^]^ The prepared thin films were found to be denser and smooth as compared with ones prepared with conventional 1‐step solution processability. Moreover, solar‐cell devices showed an improvement of ≈25% (from 0.548 to 0.686 V) in the open‐circuit voltage and 17% (from 0.070% to 0.082%) in the PCE, which was attributed to the lower charge recombination in the denser gas‐assisted PVK films. Similarly, Mali et al.^[^ ^109^ ^]^ studied the crystallization of the MBI PVKs (with a bandgap of 2.01 eV) prepared via an antisolvent strategy. Along with enhanced environmental stability, improved photovoltaic properties, i.e., PCE of 0.356% with *V*_OC_ 0.653_ _V, *J*_SC_ 1.10 mA cm^−2^, and FF of 54% were achieved, which were considerably higher than the ones prepared without antisolvent, see **Figure** **11**a,e.

#### 5.2.2 0D Bi‐Based PSCs Fabricated via Two‐Step Solution Process

Although the one‐step solution process was facile and cost‐effective but due to the rapid film crystallization, it lead toward nonuniform film morphology, more defects sites, the formation of pinholes, and less coverage of the substrate.^[^ ^55^, ^95^, ^97^, ^98^ ^]^ Furthermore, for some longer chain organic cations, it was difficult to find proper solvents. Conversely, the two‐step solution process overcame most of the above issues associated with a one‐step solution process, as reported previously for the Pb‐based PVKs.^[^ ^109^ ^]^ Therefore, uniform, smooth, and compact MBI films were synthesized via a novel two‐step evaporation‐spin‐coating technique (i.e., thermal evaporation of BiI_3_ and spin‐coating of MA).^[^ ^92^ ^]^ The excellent homogeneity and compactness of the pinholes‐free PVK film morphology further improved the optoelectronic properties of the solar‐cell device, e.g., PCE of 0.39% and a record open‐circuit voltage of 0.83 V were claimed. To make further advancement, a novel soaking‐assisted sequential two‐step method was introduced to fabricate highly uniform MBI thin films with superior coverage both on c‐TiO_2_ and m‐TiO_2_ substrates,^[^ ^110^ ^]^ as shown in Figure 11b–d. Therein, BiI_3_ films were first spin‐coated on the TiO_2_ substrates; which were successfully transformed (by dipping in MAI solution) into high‐quality MBI films. The effect of the improved morphology, as well as the device architecture on the performance of a solar‐cell was studied. It was realized that devices with m‐TiO_2_ showed better performance than the ones with planar structures. Along with excellent thermal and environmental stability, HTM‐free solar‐cell devices exhibited PCE of 0.054% with *V*_OC_ 0.89_ _V, *J*_SC_ 0.255 mA cm^−2^, and FF of 24%, see Figure 11f.

During film formation, the concentration of precursor and spinning speed play a vital role, which governs the crystal size and thickness of the PVK layer. For instance, at an optimum concentration of MAI in the precursor solution, the highest *V*_OC_ (0.72 V) was claimed.^[^ ^111^ ^]^ Similarly, the importance of MAI concentration in the precursor solution was realized when the MA_3_Bi_2_I_9_ compound was fabricated via interdiffusion of solution‐processed BiI_3_ and MA layers stacking on meso‐TiO_2_ substrate.^[^ ^112^ ^]^ In this method, BiI_3_ and MA films were first spin‐coated separately on the substrate and subsequently, the layers were converted into MBI PVK film via a solid‐state interdiffusion mechanism. At optimum concentrations, the film morphology, as well as the device performance was found to be superior to the ones fabricated via the conventional one‐step solution process, see Figure 11i. Finally, a solar‐cell device was fabricated that exhibited a PCE of 0.29% along with negligible hysteresis behavior, as shown in Figure 11e.

#### 5.2.3 0D Bi‐Based PSCs Fabricated via Dry Routes

**Figure 12:**
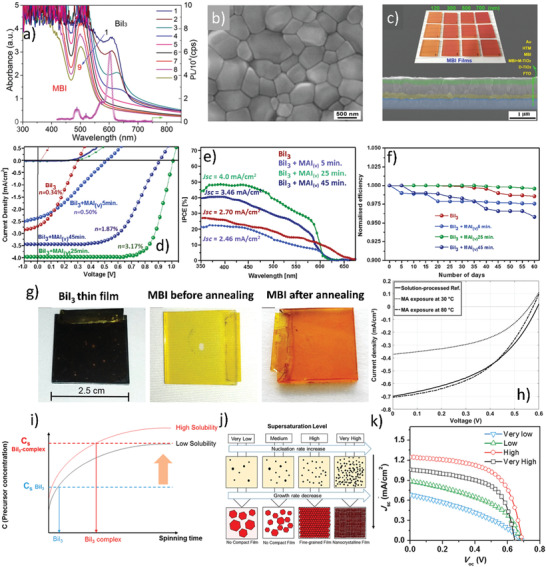
Morphological, optical, and PV properties of the MBI films fabricated via dry route and solvent‐engineering: a) Optical absorption spectra for evolution of BiI_3_ to MBI thin film processed at low vacuum with heating durations of 0, 1, 2, and 4 h at 140 °C for Samples 1–4 and 2, 4, 6, 8, and 10 h at 160 °C for samples 5–9). A PL spectrum of MBI is also shown in the same figure. b) SEM image MBI thin films. c) The cross‐sectional SEM image of the MBI‐based solar cell device. The inset images are the photographs of the MBI films with various thicknesses. Reproduced with permission.^[^ ^62b^ ^]^ Copyright 2017, American Chemical Society. d) *JV* information of the solar cell devices based on MBI thin films prepared at different exposure/reaction time to MAI vapors, e) IPCE curves, and f) the device stability of the solar cells prepared from MBI films at different reaction/exposure time to the MAI vapors. Reproduced with permission.^[^ ^113^ ^]^ Copyright 2018, Elsevier. g) The appearance of a thin film with an as‐deposited BiI_3_, intermediate state, i.e., MAI just adsorbed on BiI_3_ film (before the annealing step was performed), and MBI film after thermal annealing and exposure to air. h) Comparative *JV* characteristics of the solar cell devices fabricated via solution processing and Chemical vapor deposition (CVD) route. Reproduced with permission.^[^ ^104^ ^]^ Copyright 2019, Wiley‐VCH. i) Effect of BiI_3_ concentration and spinning time on the crystallization of MBI. Cs is the concentration at which supersaturation has occurred. j) A schematic of the film evolution versus BiI_3_ concentration and spinning time. k) Corresponding *J*–*V* characteristics. Reproduced with permission.^[^ ^118^ ^]^ Copyright 2017, American Chemical Society.

With both one‐step and two‐step solution processibilities, so far, the PCE obtained for MBI‐based devices was in the range of 0.01–0.42%. One of the possible reasons for this lower performance was the erosion of the precursor material (i.e., BiI_3_ and MAI) due to a subsequent encounter with commonly used corrosive solvents. Therefore, a solvent contact‐free two‐step solid‐state approach was adopted;^[^ ^62b^ ^]^ where BiI_3_ film was first deposited on the TiO_2_ via a high vacuum evaporator and subsequently transformed into a high‐quality MBI film in the MAI thermal atmosphere for 6–10 h, see Equation (2)^[^ ^104^ ^]^ and **Figure** **12**a–c.

(2) $$2\mathrm{Bi}I_{3}+3CH_{3}NH_{3}I\to {\left(CH_{3}NH_{3}\right)}_{3}\mathrm{Bi}I_{9}$$

To convert the leftover precursors into PVK structure, an additional step of thermal annealing was performed. Although this technique was time‐consuming and expensive, superior quality thin films with larger grain sizes were obtained. Consequently, a solar‐cell device showed a remarkably improved PCE of 1.64%, which was the highest PCE ever reported for any MBI‐based solar‐cells. Progress has been made by Jain et al.^[^ ^113^ ^]^ and applied a similar technique where a mesoporous BiI_3_ thin film was transformed into MBI by exposure to the MAI vapors. The conversion of the BiI_3_ film into a compact MBI PVK film was carefully monitored by a controlled vapor‐assisted solution process. It was observed that longer processing time is beneficial for better device performance. Finally, a solar‐cell device delivered a record PCE of 3.17% along with enhanced stability and negligible hysteresis, which is still the highest PCE ever demonstrated by phase‐pure MBI‐based solar‐cell, see Figure 12d–f. The tremendous improvement in the PCE was accredited to the improved crystallization, reduced metallic defects sites, and greater substrate coverage that avoided the shunting and recombination losses in the device.

Chemical vapor deposition (CVD) is a powerful route to fabricate high‐quality thin film and has been extensively applied for the iconic Pb‐based PVK thin films.^[^ ^114^ ^]^ Therefore, Stümmler et al.^[^ ^104^ ^]^ synthesized MBI thins films via the CVD process, where BiI_3_ spin‐coated films were exposed to the MAI vapor for transformation into MBI PVK phase, see Figure 12g,h. Furthermore, a mechanism of PVK film formation was discussed in details and a new two‐step reaction was introduced: i) adsorption of MAI and ii) the water absorption from the environment, see Equation (3)

(3) $$6\mathrm{Bi}I_{3}+6CH_{3}NH_{2}+3H_{2}O\to 2{\left(CH_{3}NH_{3}\right)}_{3}\mathrm{Bi}I_{9}+Bi_{2}O_{3}$$

In comparison with other conventional solid‐state routes, the reaction was extremely fast, occurred at lower temperatures (80 °C) and ambient conditions. Finally, solar‐cell devices prepared by this method showed comparable performance with ones fabricated via conventional solution processability. This method provided certain advantages over the others; for example, less time consumption and large‐area device fabrication.^[^ ^106^ ^]^ Therein first, the Cu–Bi alloy was sputtered on the ITO‐substrate and subsequently exposed to the vaporous I_2_ in a sealed container under a nitrogen atmosphere for 10 h, which yielded a highly oriented blackish‐gray film with a bandgap of 1.81 eV. Apart from these, comparable photoelectric characteristics with MAPbI_3_ were revealed by photoinduced carrier dynamics. Finally, a solar‐cell device demonstrated a PCE of ≈1.12%, *V*_OC_ 0.37 V, *J*_SC_ 7.18 mA cm^−2^, and FF of 28%, which was claimed to be the highest PV performance for CuBiI_4_. Similar PVK compounds with Ag instead of Cu (i.e., AgBi*_x_*I_3_ *_x_*_+1_) were fabricated via simple solution processability by Shao et al.^[^ ^115^ ^]^ that showed a maximum PCE of 0.78%.

#### 5.2.4 0D Bi‐Based PSCs Fabricated via Solvent Engineering

One of the reasons for the poor PVK film quality was the rapid evaporation of the highly volatile solvents, e.g., dimethylformamide (DMF), which was commonly exercised for the synthesis of PVK precursors. Solvent‐engineering is still a promising way to suppress the rapid evaporation of highly volatile solvents. This technique has been utilized successfully for the fabrication of high‐quality Pb‐based PVK films.^[^ ^116^ ^]^ In the solvent‐engineering technique, a less volatile solvent (e.g., dimethyl‐sulfoxide or DMSO) was combined with the highly volatile solvent (e.g., DMF). As a result, the quality of PVK thin film was improved, i.e., it first formed a PbI_2_·DMSO complexes, which was converted into high‐quality PVK crystals by subsequent annealing. Therefore, the state‐of‐the‐art PSCs with PCE > 20% are mostly fabricated through solvent‐engineering and antisolvent techniques.^[^ ^117^ ^]^ In these techniques, an antisolvent, e.g., toluene, chlorobenzene, or chloroform, etc. was poured immediately on the already spinning PVK film. As a result, the solubility of the solute was reduced and the supersaturation condition was achieved, which accelerated the nucleation process and lead toward smaller grain size and high‐quality uniform film. Therefore, Shin et al.^[^ ^118^ ^]^ utilized both of the above strategies to fabricate high‐quality PVK thin films of MBI and FA_3_Bi_2_I_9_ (FBI). In order to do that, either DMSO or butylpyridine (tBP) was combined with DMF and were used as a solvent to form bismuth complexes. It was realized that by increasing the solubility of BiI_3_, and suppressing the rapid evaporations of the solvents; the Bi‐complexes promoted the supersaturation during spinning, which lead toward highly compact PVK films, as shown in Figure 12i–k. Finally, solar‐cell devices composed of both FBI and MBI were fabricated and MBI was realized to be more suitable for PV applications that exhibited PCE of 0.71%, with *V*_OC_ 0.85 V, and FF of 73%.

Similarly, a minute amount of *N*‐methyl‐2pyrrolidone (NMP) was added into the precursor solution of MBI (in DMF).^[^ ^119^ ^]^ After the analysis of various concentrations in the precursor solution of MBI, NMP was realized as a morphology controller that governed the crystal growth of MBI film. With the optimized amount of NMP (25 mL) in the precursor solution, the *J*_SC_ of a solar‐cell was successfully improved by 50% as compared with devices prepared without NMP. Along with enhanced environmental stability and high reproducibility, the solar‐cell devices yielded a PCE of 0.31% with *V*_OC_ 0.51_ _V, *J*_SC_ 0.94 mA cm^−2^, and FF of 61%.

Besides the aforementioned film fabrication techniques, other techniques were also applied. For instance, a solvent annealing technique was applied to fabricate larger grain‐sized, compact CBI and Cs_3_Bi_2_I_10_ films with bandgaps of 2.08 and 1.80 eV, respectively.^[^ ^120^ ^]^ Therein, an antisolvent dripped film was annealed under the DMF vaporous atmosphere to prevent the rapid evaporation of the DMF from the precursor, which lead toward a homogenous and pinhole‐free PVK films. Moreover, interfacial engineering was also applied to eliminate the interface band‐offset by utilizing PTAA and NiO*_x_* as hole transporting layers (HTLs). Consequently, a solar‐cell made of CBI (with NiO*_x_* as HTL) demonstrated better PV performance, i.e., a PCE of 1.26% with *V*_OC_ 0.61_ _V, *J*_SC_ 1.12 mA cm^−2^, and FF of 43%. The improved PV performance was attributed to the better film morphology, elimination of band offset, and control over the leftover metal contents in the film. Similarly, ultrathin all inorganic CBI nanosheets were fabricated via the dissolution recrystallization process.^[^ ^121^ ^]^ It was revealed by scanning electron and atomic force microscopy that nanosheets formed a uniform and compact thin film with a lower surface roughness of 4.3 nm, which could be beneficial for better contact with charge transporting layer. Consequently, along with better environmental stability, a solar‐cell device demonstrated a significantly higher PCE of 3.20% along with *V*_OC_ 0.86 V, *J*_SC_ 5.78 mA cm^−2^, and FF of 64.38%. Up to the best of our knowledge, it was the highest PCE ever reported for the 0D Bi‐based PVKs.

From the above discussion, it can be concluded that compositional and interfacial engineering along with antisolvent dripping is the most promising way, which is not only cost‐effective and facile but also less time consuming for the fabrication of high‐quality Bi‐based PVK films and high‐performance PV devices.

### 5.3 Performance of the 0D Bi‐Based PSCs Synthesized in Green Solvents

In addition to Pb, the consumption of less volatile toxic organic solvents (e.g., DMF and DMSO) in large amount are also hazardous for humans and their environment. Therefore, Li et al.^[^ ^122^ ^]^ successfully synthesized the MBI perovskite compound in the green solvent, i.e., ethanol. As compared with the typical toxic organic solvents (DMF and DMSO), MA‐BiI_3_ complexes showed a higher solubility in ethanol; therefore, PVK films showed a denser and homogenous morphology. Consequently, enhanced photovoltaic properties, i.e., a PCE of 0.022% with significantly improved open‐circuit voltage of 0.84 V and *J*_SC_ 0.17 mA cm^−2^ were obtained. Similarly, methyl acetate was used as a nontoxic solvent for the lead‐free MBI PVK and high‐quality uniform thin films were fabricated on meso‐TiO_2_ for solar‐cell applications.^[^ ^123^ ^]^ After fine‐tuning of the HTL, a solar‐cell device yielded a PCE of 1.62% under 1 sun illumination. Moreover, a solar‐cell device demonstrated an enhanced photostability of 300 h under 1 sun illumination without any UV shutter.

### 5.4 Bi‐Based Double PVKs Solar Cells

**Figure 13:**
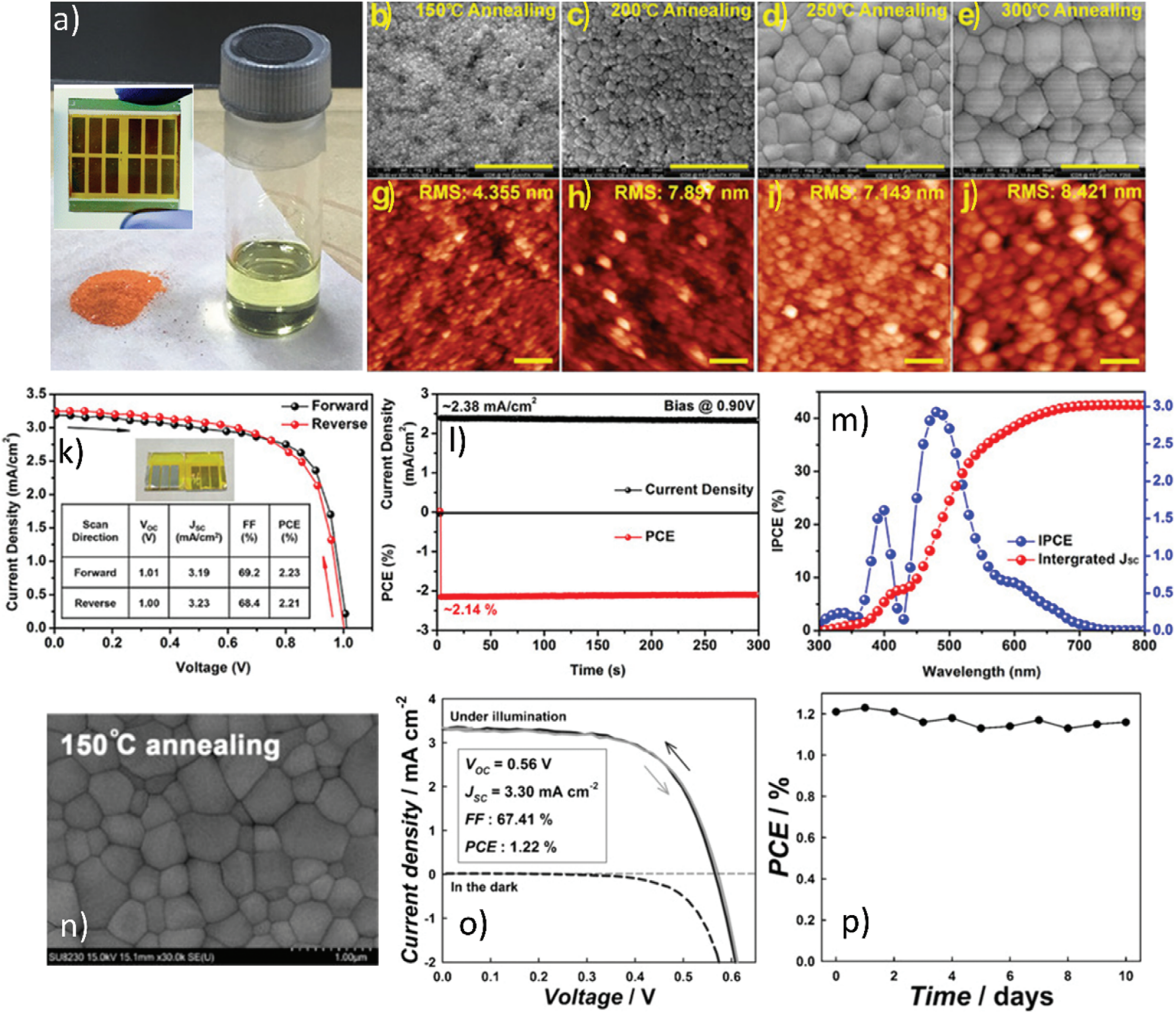
Morphological and PV characteristics of double PVKs and Rudorffites: a) A photograph of the double PVK (Cs_2_AgBiBr_6_) powder, solution in DMSO, and device module. Reproduced with permission.^[^ ^126^ ^]^ Copyright 2017, The Royal Society of Chemistry and 2017, Wiley‐VCH. b–j) SEM and AFM images of films prepared at different temperatures. k,l) *J–V* performance (forward and reverse scans) and stabilized power output along with current–density (measured under 1 sun illumination). m) IPCE‐spectra‐integrated *J*_SC_ for the best‐performed devices. Reproduced with permission.^[^ ^128^ ^]^ Copyright 2018, Wiley‐VCH. n) SEM image of the Rudorffite (AgBi_2_I_7_). o,p) are its *JV*‐characteristics and stability results. Reproduced with permission.^[^ ^130^ ^]^ Copyright 2018, Wiley‐VCH.

One of the reasons for the poor PV performance of 0D A_3_Bi_2_X_9_ PVKs was their lower dimensionality, which rendered them to high exciton binding energy, shorter carrier lifetime, higher charge trapping density, and lower charge carrier mobilities. Indeed, the higher charge of Bi^3+^ made it harder to adapt a typical 3D‐PVK structure (e.g., ABX_3_).^[^ ^41^, ^43^ ^]^ In order to incorporate Bi^3+^ into PVK framework such that it maintains a 3D structure, a class of quaternary (A_2_M^+^M^3+^X_6_) PVKs known as “elpasolites” or “double PVKs” was introduced, which could extend the conventional 0D A_3_Bi_2_X_9_ PVKs into 3D ones, unaffecting the total number of valence electrons. Initially, several double PVK compounds with numerous combinations of M^+^ and M^3+^ metal cations were suggested. Although after many theoretical studies, it was realized that only compounds with proper enthalpy, energy bandgap, exciton binding energy, and carrier effective masses could be utilized for PV applications.^[^ ^86^, ^124^ ^]^ Among those PVKs, Cs_2_BiAgBr_6_ was suggested to be the most suitable one for PV applications due to its extraordinary properties including, lower bandgap, higher stability, extended carrier lifetime, and comparable carrier effective masses.^[^ ^85^, ^86^, ^124^, ^125^ ^]^ Despite these remarkable properties, the lower solubility of Cs_2_BiAgBr_6_ precursors was hindering it to form a compact and uniform PVK thin films; until an effort was made by Greul et al.^[^ ^126^ ^]^ to fabricate Cs_2_BiAgBr_6_ thin films and utilized them successfully into a working solar‐cell device. After optimization, a solar‐cell device showed a considerable PCE of 2.5% with a significantly high open‐circuit voltage of over 1 V, which was claimed to be the highest *V*_OC_ to date for any Bi‐based PVK solar‐cell. Moreover, the solar‐cell device showed improved stability in operational conditions. Subsequently, a first lead‐free heterojunction PVK solar‐cell composed of high‐quality double‐PVK (i.e., Cs_2_AgBiBr_6_) was fabricated through a low pressure‐assisted solution process in an ambient environment.^[^ ^127^ ^]^ It was observed that the annealing temperature had a pronounced effect on the film quality, i.e., the film quality was significantly improved when the temperature varied from 150 to 300 °C. Finally, an unencapsulated device not only showed better resistance to the ambient environment but also exhibited an improved PV performance, i.e., a PCE of 1.44% along with *V*_OC_ 0.99_ _V, *J*_SC_ 1.79 mA cm^−2^ and FF of 65%. Recently, the same double PVK was utilized into a planar heterojunction solar‐cell and achieved a significantly improved PCE of 2.23% with *V*_OC_ 1.10_ _V, *J*_SC_ 3.19 mA cm^−2^ and FF of 69.2%,^[^ ^128^ ^]^ see **Figure** **13**a–m. The improved device performance was ascribed to the ultrasmooth morphology and bigger crystallite size, achieved via antisolvent treatment and high‐temperature post‐annealing. In addition, Cs_2_AgBiBr_6_, Zhang et al.^[^ ^38^ ^]^ introduced another novel double PVK, i.e., Cs_2_BiNaI_6_ via a hydrothermal process. The proposed PVK compound showed a relatively smaller bandgap of 1.66 eV; however, besides improved environmental stability, a solar‐cell device made of this PVK compound delivered poor performance, i.e., PCE of 0.42% with *V*_OC_ 0.47_ _V, *J*_SC_ 1.99 mA cm^−2^ and FF of 44%.

Although in comparison with 0D MBI PVKs, its 3D‐counterparts (i.e., double PVKs) demonstrated some improvement in the PCE along with better short‐term stability, still their PCE could not exceed over 2.5%. The possible reasons for this lower PV performance could be: i) a wider and indirect energy bandgap (i.e., 2–3 eV), ii) the presence of Ag, which was degraded after long term exposure to ambient conditions,^[^ ^124a^ ^]^ and iii) the inclusion of the monovalent cation that restricted the combination of different A and B‐site cations to achieve the desired PV properties.^[^ ^86b^ ^]^

To address these issues and establish a stable 3D Bi‐based PVK framework with lower bandgap, a family of double PVK oxides with general formula A′A″B′BiO_6_ was studied. Such a novel structure provided a possibility of a vast combination of A‐site cations and tunable bandgaps. For instance, a double PVK with triple A‐site cations, i.e., KBaTeBiI_6_ with an optical bandgap of 1.88 eV was reported by Thind et al.^[^ ^129^ ^]^ The proposed structure was first modeled theoretically and afterward, it was fabricated via a wet chemical approach. It was realized that the effective masses of the charge carriers were comparable to those of the best performance Bi‐based double PVKs materials. Along with better environmental stability, a solar‐cell device exhibited PCE of 0.057% with *V*_OC_ 0.54_ _V, *J*_SC_ 0.09 mA cm^−2^ and FF of 58%. The poor PV performance of a device was attributed to the stoichiometric and structural defects.

### 5.5 Lead‐Free Rudorffites‐Based PSCs

Among various families of Bi‐based PVKs, the silver bismuth iodide based PVKs showed a suitable bandgap (1.62–1.8 eV) for the PV applications.^[^ ^17^, ^130^, ^131^ ^]^ Therefore, silver bismuth iodide compounds with various stoichiometries, i.e., A*_a_*B*_b_*X*_x_* (with *x* = *a* + 3*b*) were introduced. This family (included compounds like Ag_3_BiI_6_, Ag_2_BiI_5_, AgBiI_4_, and AgBi_2_I_7_, etc.) was named as “Rudorffites;” after Walter Rudorff, the discoverer of the similar prototype oxides, i.e., NaVO_2_.

A first PV application of the silver bismuth iodide PVK compound, i.e., AgBi_2_I_7_ with pure cubic structure (i.e., *Fd*_3_ *m*, revealed by X‐ray diffraction (XRD) analysis) was presented by Kim et al.^[^ ^130^ ^]^ The solution‐processed spin‐coated film of AgBi_2_I_7_ exhibited a dense pinhole‐free morphology and a relatively lower bandgap of 1.87 eV, suitable for photovoltaic applications. Moreover, it was observed that the phase‐pure cubic AgBi_2_I_7_ compound can only be obtained at temperatures > 110 °C, as shown in Figure 13n. Finally, a solar‐cell device demonstrated a PCE of 1.22% with *V*_OC_ 0.56 V, *J*_SC_ 3.30 mA cm^−2^, and FF of 67.41%, which was well preserved for 10 days in the ambient environment, see Figure 13o,p. A similar PVK (i.e., AgBi_2_I_7_) with a hexagonal structure and indirect (1.62 eV) and direct (1.85 eV) bandgaps were also reported.^[^ ^131c^ ^]^ A photocurrent in a broad range of EM‐spectrum (i.e., 350–700 nm) with a maximum external quantum yield of 45% was recorded. Furthermore, solar‐cells made of this PVK material yielded an improved photovoltaic performance, i.e., a PCE of 2.1% with *V*_OC_ 0.49 V, *J*_SC_ 6.8 mA cm^−2^. Likewise, a step toward further improvement in the performance of Rudorffites‐based PSCs was taken by Turkevych et al.^[^ ^17a^ ^]^ and introduced the same compound (AgBi_2_I_7_ with cubic structure) along with other silver bismuth iodides PVKs (i.e., Ag_3_BiI_6_, Ag_2_BiI_5_, and AgBiI_4_ with bandgaps ranging 1.79–1.83 eV). A solar‐cell device with configuration (FTO/c‐m‐TiO_2_/Ag_3_BiI_6_/PTAA/Au) demonstrated significantly improved PCE of 4.3% along with *V*_OC_ 0.63 V, *J*_SC_ 10.7 mA cm^−2^, and FF of 64%. This was the best PV performance to date exhibited by any Bi‐based PVK solar‐cell. Successively, a Rudorffite compound, i.e., Ag_2_BiI_5_ with various BiI_3_ to AgI ratios was synthesized by solid‐state reaction.^[^ ^132^ ^]^ A solar‐cell device with pure Ag_2_BiI_5_ exhibited PCE of 1.74% whereas a device with an excessive amount of BiI_3_ (as an impurity) yielded significantly improved PV‐characteristics, i.e., a PCE of 2.31%, *V*_OC_ 0.594 V, *J*_SC_ 4.20 mA cm^−2^, and FF of 54.8%. Moreover, along with negligible hysteresis, a solar‐cell device showed a long‐term resistance to environmental influences. It is to be noted that even though Turkevych et al.^[^ ^17a^ ^]^ and Jung et al.^[^ ^132^ ^]^ achieved a considerably improved PCE along with better stability, their synthesis routes were very expensive in terms of temperature (>500 °C) and time‐consumption; therefore, could not be applied on large scale. Recently, a two‐step coevaporation or annealing technique was utilized to fabricate thin films of AgBiI_4_, Ag_2_BiI_5_, and Ag_2_BiI_7_ with improved surface morphology and larger crystal size (>3 µm).^[^ ^105^ ^]^ A solar‐cell device based on these compounds demonstrated a relatively lower PCE of 0.9% and improved *V*_OC_ of > 0.8 V. Unfortunately, along with underperformed PCE, a solar‐cell device displayed a significantly larger hysteresis loss, which could be attributed to the higher defects concentration in the material.

The grain size for the PVK film is a critical parameter that governs the PV performance of a device. For instance, a direct relationship between the size of the PVK crystal and photocurrent was reported.^[^ ^133^ ^]^ One way to fabricate thin films with larger grain size is hot casting, i.e., spinning or pouring the PVK precursor solution on a hot substrate.^[^ ^134^ ^]^ Therefore, Gosh et al.^[^ ^135^ ^]^ utilized a dynamic hot‐casting technique to fabricate complex 3D analogs of ternary bismuth halide PVKs (i.e., AgBiI_4_ and AgBiI_5_) and achieved compact PVK films with considerably large grain sizes. Solar cells with mesoscopic architectures were fabricated in the ambient environment (RH  ≥  65%), which yielded a record PCE (i.e., 2.2% and 2.62% for AgBiI_4_ and AgBiI_5_, respectively) among all solution‐processed Bi‐based PVKs. It should be noted that Turkevych et al.^[^ ^17a^ ^]^ reported a PCE of 4.3% for the similar PVK (AgBiI_6_) but that was synthesized via expensive solid‐state routes with processing temperature higher than 500 °C.

### 5.6 Bi‐Based PSCs with New Cations

In addition to the above Bi‐based compounds where MA, Cs, FA, and Ag were used as A‐site cations, structures with new organic cations were also applied for improved PV performance. For example, Fabian et al.^[^ ^46^ ^]^ introduced a corrugated 1D hybrid organic–inorganic Bi‐based n‐type PVK compound (i.e., HDABiI_5_) with an optical bandgap of ≈2.1 eV. A photoelectrochemical analysis revealed that HDABiI_5_ based devices demonstrated a steady‐state short‐circuit current density of ≈100 µA cm^−2^ along with a stable *V*_OC_ of 400 mV under 1 sun illumination. Furthermore, along with better stability, a solar‐cell device made of such a PVK compound exhibited a PCE of 0.027% with *V*_OC_ 0.43_ _V and *J*_SC_ 0.124 mA cm^−2^.

Similarly, two novel organic–inorganic Bi‐based PVKs (i.e., C_5_H_6_NBiI_4_ and C_6_H_8_NBiI_4_) with a bandgap of 2.0 eV and 1D structure (formed by edge‐shared octahedra) were reported.^[^ ^136^ ^]^ It was revealed by density functional theory (DFT) calculations that the organic entities in the structure promoted the intermolecular interaction, which supported the ability of pseudo‐3D charge transport due to matching energies with the conduction band minima. Along with better environmental stability, a solar‐cell device (without HTL) yielded a PCE of 0.9% with *V*_OC_ 0.62_ _V, *J*_SC_ 2.71 mA cm^−2^, and FF of 54%. Apart from this, three novel organic–inorganic Bi‐based PVKs, i.e., (C_3_H_4_NS)_3_Bi_2_I_9_, (C_3_H_4_N_2_)_3_Bi_2_I_9_, and (C_3_H_5_N_2_S)BiI_4_, were introduced.^[^ ^137^ ^]^ The first two PVKs were found to be 0D while the third one was 1D with optical bandgaps of 2.08, 2.0, and 1.78 eV, respectively. Among these PVKs, the best performance solar‐cell based on (C_3_H_5_N_2_S)BiI_4_ delivered a PCE of 0.47%.

### 5.7 Sulfur and Bi‐Based PSCs

**Figure 14:**
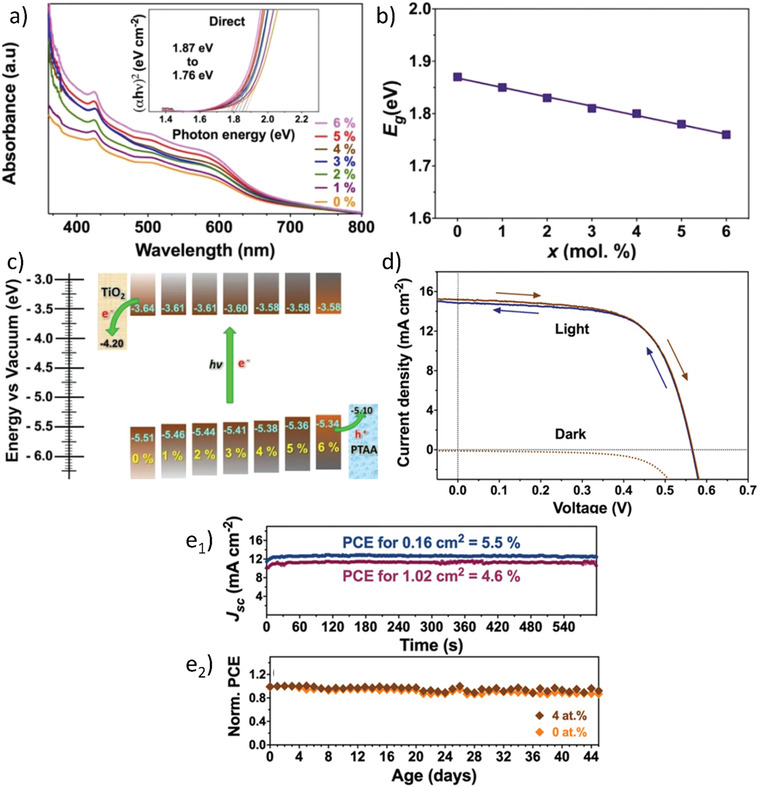
Optoelectronic properties and device characteristics of the Ag, Bi, and S based PSCs: a) Absorption spectra and Tauc plots (inset) for *x* = 0–6%, where *x* is the concentration of sulfur. b) Variation in the optical bandgap with the increasing amount of anionic sulfide substitution; and c) the electronic structure at different *x* (energy of the conduction band edge for TiO_2_ and of the HOMO level for PTAA are provided for comparison. d) Comparative JV curves in both reverse and forward directions (recorded in dark and light with sweep rate 100 mV s^−1^). e_1_) Photocurrent transients recorded at potentials of maximal power derived from *J*–*V* data for the devices tested with an aperture of 0.16 cm^2^ at 431 mV (navy) and an aperture of 1.02 cm^2^ at 409 mV (purple); e_2_) evolution of normalized PCE of the solar‐cells (aperture 0.16 cm^2^) with *x* = 0 (orange) and 4 at% (brown) stored in air under diffuse light. Reproduced with permission.^[^ ^138^ ^]^ Copyright 2018, Wiley‐VCH.

Although silver bismuth iodides (Ag*_a_*Bi*_b_*I*_a_*_+3_ *_b_*) exhibited better PV performance among the other Bi‐based PVKs, still their PCE was far lower. The reason for this lower PCE was their wide bandgap and downshifted valence band edges. To address these issues, a sulfide dianion was utilized as an anionic substitute, which successfully altered the optoelectronic properties of Ag_a_Bi_b_I_a+3b_ based PVKs.^[^ ^138^ ^]^ With this strategy, the energy bandgap was successfully narrowed by 0.1 eV where the valence band edge was lifted up by 0.1–0.3 eV in the different stoichiometries of silver bismuth iodides (i.e., AgBiI_4_, Ag_2_BiI_5_, Ag_3_BiI_6_, and AgBi_2_I_7_). Solar‐cell devices based on these silver bismuth sulfoiodides presented a remarkable improvement in the device performance and PCE was boosted up to more than 5.44%, along with *V*_OC_ = 0.57 V, *J*_SC_ 14.6 ± 0.1 mA cm^−2^, and FF of 66%, see **Figure** **14**a–e. So far these are the best PV characteristics for any Bi‐based lead‐free PVK solar‐cell. Similarly, AgBiS_2_ nanocrystals (dispersible in most of the organic solvents) were synthesized via the hot‐injection method.^[^ ^139^ ^]^ The nanocrystals exhibited a strong absorption with absorption coefficient ≈10^3^–10^5^ cm^−1^, a suitable bandgap of 1.3 eV, and excellent environmental stability. Finally, the optimized device based on tetramethylammonium iodide (TMAI)‐treated AgBiS_2_ solar‐cells demonstrated the best performance with a PTB7 as an HTL and yielded a certified PCE of 6.3%. To the best of our knowledge, this is the highest PCE reported for any Bi‐based PSCs yet.

Additionally, Bi is also used in a combination with other lower‐dimensional material to form alloyed structures and enhance PV performance. For instance, it was combined with quasi‐1D SbSI to form an alloyed compound, i.e., Sb_1−_ *_x_*Bi*_x_*SI where halides and chalcogenides coexisted.^[^ ^140^ ^]^ The alloyed compound exhibited a bandgap of 1.62 eV, which was suitable for photovoltaic applications. Under standard illuminations, a solar‐cell device based on this compound exhibited a better PCE of 4.07%, which was retained up to 92% under standard conditions with a relative humidity of 60% and a temperature of 85 °C for 360 h. It is important to note that the SbSI compound (without Bi) with a bandgap of 2.1 eV was previously used for PV application where a PCE of 3.05% was recorded. Similarly, another novel PVK compound (i.e., MABiI_2_S) based on the halide‐chalcogenides combination was reported, which exhibited even a lower bandgap of 1.52 eV, more suitable for the PV devices.^[^ ^141^ ^]^ Yet, a solar‐cell device made of this compound showed a relatively lower PCE of 0.13%.

### 5.8 Bi‐Based PSCs with Bi at A‐Site

Besides, being a B‐site element of the PVK architecture, Bi has also been utilized at A‐site along with other similar materials. For instance, it was used with Mn (at the A‐site of the PVK framework) to fabricate a multifunctional PV device based on the composite of the mixed phases (BiMnO_3_ and BiMnO_5_).^[^ ^142^ ^]^ A considerable PCE of 4.2% along with *V*_OC_ 1.48 V, *J*_SC_ 7.03 mA cm^−2^, and FF of 58% was obtained under 1 sun illumination. It was understood that instead of the interior of the crystal grains, the photocurrent was developed at the grain boundaries. Moreover, the *V*_OC_ and *J*_SC_ were found to be governed by the resistance of the device, hence showed the multifunctionality of the photovoltaic device. It is noteworthy that unlike the above work, the performance of the semiconducting devices usually deteriorates upon turning the material from a single crystal to polycrystalline. However, as a benefit of the combination of the two oxide phases, it was recognized that the resultant *V*_OC_ was greater than that of the individual compounds, hence high‐performance solar‐cell device was obtained.

**Figure 15:**
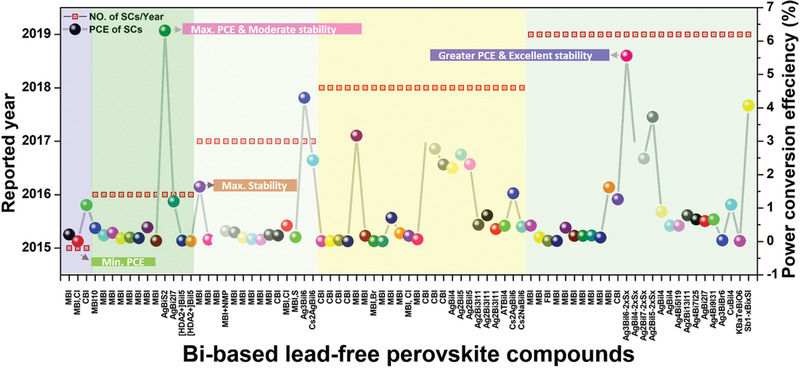
The overall record of Bi‐based perovskites solar cell along with the compound name and recorded PCE. The better performance devices on the basis of the power conversion efficiency and stability are labeled. The data was collected from Web of Science by searching the keywords “bismuth + perovskites + solar cells.”

From all of the above discussion about the photovoltaic applications of the Bi‐based PVKs, it can be concluded that better film morphology, lower energy bandgap, and minimum energy band offset (between the active layer and HTM) could enable the Bi‐based PSCs to achieve an enhanced PCE and better environmental stability. So far, these requirements are mostly fulfilled by the strategy of alloying or doping of other lower bandgap material (e.g., sulfur) with Bi; boosted the PCE above 6.3%, which was under 4% for the phase‐pure Bi‐based PSCs. The overall progress of the reported Bi‐based PSCs is summarized in **Table** **1** and **Figure** **15**.

**Table 1:** The overall record of the Bi‐based perovskites solar cells along with the compound name, device architecture, PV‐characteristic, optical bandgap, reported stability and method of testing, and publishing year. The data for the table was collected from the Web of Science by searching the keywords, “bismuth + perovskites + solar cell”

Perovskite	Device architecture	*V*_OC_ [V]	*J*_SC_ [mA m^−2^]	FF [%]	PCE [%]	*E*_BG_ [eV]	Device stability and testing method	Year	Ref.
0D									
A_3_Bi_2_X_9_									
MA_3_Bi_2_I_9_	Ag/Spiro/PVK/c‐TiO_2_/FTO	0.52	0.68	33	0.120	2.10	H_r_10%,>1 month, retained PCE ≈100%	2015	^[^ ^55^ ^]^
MA_3_Bi_2_I_9_Cl*_x_*		0.04	0.18	38	0.003	2.40			
Cs_3_Bi_2_I_9_		0.85	2.15	60	1.090	2.20			
MA_3_Bi_2_I_9_	Au/P3HT/PVK/TiO_2_	0.35	1.16	46	0.190	1.74	H_r_ > 50%, 21 days, retained PCE ≈100%	2016	^[^ ^95^ ^]^
MA_3_Bi_2_I_9_	Au/HTM/PVK/Anatase/FTO	0.56	0.83	48	0.259	–	Ambient, 10 weeks, retained PCE ≈75%	2016	^[^ ^99^ ^]^
MA_3_Bi_2_I_9_	Au/HTM/PVK/Brookite/FTO	0.53	0.57	30	0.094	–			
MA_3_Bi_2_I_9_	Ag/Spiro/MoO_3_/PVK/c‐TiO_2_/ITO	0.59–063	0.35–0.44	34	0.10–0.12	2.05	H_r_ 50%, 21 days, retained PCE ≈100%	2016	^[^ ^100^ ^]^
	Ag/Spiro/MoO_3_/PVK/m‐TiO_2_/ITO	0.47–0.59	0.20–0.47	47–52	0.06–0.42				
MA_3_Bi_2_I_9_	Al/Ca/PCBM/PVK/PEDOT/ITO	0.66	0.22	0.49	0.100	2.94	Ambient, several months, film color unchanged	2016	^[^ ^98^ ^]^
MA_3_Bi_2_I_9_	Ag/P3HT/PVK/TiO_2_/FTO	0.26	0.18	37	0.020	2.03	Ambient, 17 h, retained PCE ≈100%	2016	^[^ ^101^ ^]^
MA_3_Bi_2_I_9_	Au/Spiro/PVK/TiO_2_/FTO	0.81	2.95	69	1.640	2.26	H_r_ 40–70%, 13 weeks, retained PCE ≈100%	2017	^[^ ^62b^ ^]^
MA_3_Bi_2_I_9_	C/PVK/m‐TiO_2_/FTO	0.89	0.25	24	0.054	2.17	Hr 40%, 201 days, film structure almost unchanged revealed by XRD analysis. retained PCE ≈100% for 32 days	2017	^[^ ^110^ ^]^
	C/PVK/c‐TiO_2_/FTO	0.75	0.23	23	0.039				
MA_3_Bi_2_I_9_ +NMP	Au/Spiro/PVK/m‐TiO_2_/c‐TiO_2_/FTO	0.51	0.94	61	0.31	–	Hr 30–60%, 31 days, retained PCE ≈100%	2017	^[^ ^119^ ^]^
MA_3_Bi_2_I_9_	Au/Spiro/PVK/TiO_2_/FTO	0.62	1.16	49	0.270	–	–	2017	^[^ ^112^ ^]^
MA_3_Bi_2_I_9_	Au/Spiro/PVK/TiO_2_/ITO	0.72	0.49	32	0.110	1.96	Ambient, Hr 50–60%, 30 days film color unchanged	2017	^[^ ^97^ ^]^
MA_3_Bi_2_I_9_	Au/Spiro/PVK/TiO_2_/FTO	0.65	1.10	50	0.069	2.10	Ambient, 60 days, retained PCE >70%	2017	^[110]^
MA_3_Bi_2_I_9_	Au/Spiro/PVK/c‐TiO_2_/FTO	0.84	0.17	35	0.053	–	Ambient, Hr 50%, 5 days, retained PCE ≈100%	2017	^[^ ^122^ ^]^
MA_3_Bi_2_I_9_	Au/Spiro/PVK/c‐TiO_2_/FTO	0.53	0.65	57	0.200	1.90	Ambient, 2 months, film structure almost unchanged revealed by XRD analysis	2017	^[^ ^96^ ^]^
Cs_3_Bi_2_I_9_	Au/Spiro/PVK/c‐TiO_2_/FTO	0.54	0.58	57	0.180	1.90	Ambient, 2 months, film structure almost unchanged revealed by XRD analysis	2017	^[^ ^96^ ^]^
Cs_3_Bi_2_I_9_	Au/Spiro/PVK/m‐TiO_2_/c‐TiO_2_/FTO	0.52	0.04	45	0.009	2.00	–	2018	^[^ ^43d^ ^]^
	Au/PolyTPD/PVK/m‐TiO_2_/c‐TiO_2_/FTO	0.32	0.12	35	0.013				
	Au/PTAA/PVK/m‐TiO_2_/c‐TiO_2_/FTO	0.53	0.11	56	0.034				
	Au/PVK/m‐TiO_2_/c‐TiO_2_/FTO	0.69	0.15	28	0.003				
MA_3_Bi_2_I_9_	Au/P3HT/PVK/c‐TiO_2_/FTO	1.01	4.02	78	3.170	1.90	Ambient, 60 days, retained PCE ≈100%	2018	^[^ ^123^ ^]^
MA_3_Bi_2_I_9_	Au/PVK/m‐TiO_2_/c‐TiO_2_/FTO	0.72	0.61	38	0.169	–	Ambient, 11 days, retained PCE ≈54%	2018	^[^ ^111^ ^]^
FA_3_Bi_2_I_9_	Au/Spiro/PVK/m‐TiO_2_/c‐TiO_2_/ITO	0.45	1.10	46	0.022	2.19	–	2019	^[^ ^155^ ^]^
MA_3_Bi_2_I_9_		0.48	0.078	37	0.013	2.25			
MA_3_Bi_2_I_9_	Au/Spiro/PVK/m‐TiO_2_/c‐TiO_2_/ITO	0.87	1.60	34	0.410	2.20	Ambient, 60 days, retained PCE ≈51.2%	2019	^[^ ^103^ ^]^
MA_3_Bi_2_I_9_	Ag/P3HT/PVK/m‐TiO_2_/c‐TiO_2_/FTO	0.21	2.33	33	0.170	2.24	–	2019	^[^ ^107^ ^]^
MA_3_Bi_2_I_9_	Ag/Au/Spiro/PVK/TiO_2_/FTO	0.59	0.50	57	0.170	–	Hr 60% and 22 °C, 13 days, retained PCE 77%	2019	^[^ ^102^ ^]^
MA_3_Bi_2_I_9_	Au/Spiro/PVK/TiO_2_/FTO	0.37	0.71	–	0.176	1.97	–	2019	^[^ ^104^ ^]^
MA_3_Bi_2_I_9_	Carbon/P3HT/PVK/TiO_2_/FTO	0.76	2.75	54	1.120	2.1	1 sun illumination, 300 h, retained PCE ≈98%	2019	^[^ ^113^ ^]^
	Carbon/Spiro/PVK/TiO_2_/FTO	0.87	2.70	69	1.62				
Cs_3_Bi_2_I_9_	Ag/AZO/PCBM/PVK/PEDOT/PTAA/NiO*_x_*/ITO	0.74	3.42	51	1.26	2.08	Ambient 6 months, film structure unchanged, revealed by XRD analysis	2019	^[^ ^120^ ^]^
MA_3_Bi_2_I_9_	Au/PIF8‐TAA/PVK/m‐TiO_2_/c‐TiO_2_/FTO	0.85	1.25	85	0.710	–	Ambient air, 10 days, retained PCE 80%	2018	^[^ ^118^ ^]^
MA_3_Bi_2_I_9_	Au/Spiro/PVK/m‐TiO_2_/c‐TiO_2_/ITO	0.58	1.04	48	0.250	2.25	Inert condition, 700 days, retained PCE ≈100%	2018	^[^ ^10a^ ^]^
MA_3_Bi_2_I_9−_ *_x_*Cl*_x_*		0.45	0.66	58	0.170	2.38			
MA_3_Bi_2_I_9_	Ag/MoO_3_/Spiro/PVK/FPDI/ITO	0.61	0.37	28	0.063	–	Ambient, 17 days, retained PCE ≈100%	2018	^[^ ^156^ ^]^
AgBiI_4_	Au/PTAA/PVK/m‐TiO_2_/c‐TiO_2_/FTO	0.67	5.24	62	2.200	1.66	Hr 60–75% and 20 °C, 1 month, retained PCE ≈100%	2018	^[^ ^135^ ^]^
Ag_2_BiI_5_		0.69	6.04	62	2.600	1.70			
Ag_3_BiI_6−2_ *_x_*S*_x_*	Au/PTAA/PVK/m‐TiO_2_/c‐TiO_2_/FTO	0.57	14.7	66	5.560	–	Hr 40% and 24 °C, 45 days, retained PCE ≈90%	2019	^[^ ^138^ ^]^
AgBiI_4−2_ *_x_*S*_x_*		0.52	9.46	55	2.750	1.73	–		
Ag_2_BiI_7−2_ *_x_*S*_x_*		0.57	7.68	56.6	2.480	1.79	–		
Ag_2_BiI_5−2_ *_x_*S*_x_*		0.48	13.1	61	3.73	1.77	–		
Cs_2_NaBiI_6_	Au/Spiro/PVK/m‐TiO_2_/c‐TiO_2_/FTO	0.47	1.99	44	0.440	1.66	Ambient, 14 days, retained PCE ≈100%	2018	^[^ ^38^ ^]^
MABiI_2_S	Au/Spiro/PVK/m‐TiO2/c‐TiO_2_/FTO	0.22	1.96	30	0.13	1.50	–	2019	^[^ ^141^ ^]^
AgBiI_4_	Au/P3HT/PVK/c‐TiO_2_/FTO	0.84	2.37	44	0.890	1.80	Inert condition, 6 months, no change in film, revealed by XRD analysis	2019	^[^ ^105^ ^]^
AgBiI_4_	Au/Spiro/PVK/SnO_2_/m‐TiO_2_/c‐TiO_2_/FTO	0.62	1.82	41	0.470	1.80	Inert condition, 2 weeks, retained PCE 75%	2019	^[^ ^115^ ^]^
Ag_4_Bi_5_I_19_		0.66	1.51	47	0.470				
Ag_2_Bi_3_I_11_		0.72	2.39	46	0.780				
Ag_4_Bi_7_I_25_		0.71	2.57	36	0.660				
AgBi_2_I_7_		0.65	2.51	38	0.610				
Ag_4_Bi_9_I_31_		0.66	2.60	38	0.660				
Cs_3_Bi_2_I_9_	Au/CuI/PVK/c‐TiO_2_/FTO	0.86	5.78	64	3.200	–	Abm., 38 days, retained PCE 57%	2018	^[^ ^121^ ^]^
	Au/Spiro/PVK/c‐TiO_2_/FTO	0.79	4.45	50	2.770		Abm., 38 days, retained PCE 28%		
	Au/PTAA/PVK/c‐TiO_2_/FTO	0.83	4.82	57	2.300		Abm., 38 days, retained PCE 47%		
MA_3_Bi_2_I_9−_ *_x_*Br*_x_*	Au/Spiro/PVK/c‐TiO2/FTO	0.39	0.07	32	0.010	2.18	Ambient 132 days, film abs. peaks slightly declined	2018	^[^ ^157^ ^]^
MA_3_Bi_2_I_9_		0.48	0.05	25	0.006	2.10			
MA_3_Bi_2_I_9−_ *_x_*Cl*_x_*	–	0.022	0.07	32	0.473	1.9–2.34	–	2019	^[^ ^158^ ^]^
BiMnO_3_/BiMn_2_O_5_	STO‐single crystal/PVK/ITO	1.48	7.03	58	4.200	1.42	–	2018	^[^ ^142^ ^]^
CuBiI_4_	Au/Spiro/PVK/ITO	0.37	0.71	28	1.100	1.79	–	2019	^[^ ^106^ ^]^
Sb_1−_ *_x_*Bi*_x_*SI	Ag/PCPDTBT/PVK/m‐TiO_2_/TiO2BL/FTO	0.53	14.54	53	4.070	1.81	Hr 60%, 360 h, retained PCE 92%	2019	^[^ ^140^ ^]^
ATBiI_4_	C/ZrO_2_/ATB/PVK/THB/m‐TiO_2_/FTO	0.37	3.29	39	0.470	1.78	Ambient, dark, two weeks, film structure unchanged, revealed by XRD	2018	^[^ ^137^ ^]^
Cs_2_AgBiBr_6_	Au/P3HT/PVK/SnO_2_/ITO	1.07	1.78	69	1.440	2.05	Hr 40–60% and 20–40 °C, retained PCE ≈98%	2018	^[^ ^127^ ^]^
Ag_2_Bi_3_I_11_	Au/Spiro/PVK/m‐TiO_2_/c‐TiO_2_/FTO	0.71	1.69	39	0.500	1.52	–	2018	^[^ ^159^ ^]^
	Au/Spiro/PVK/SnO_2_/FTO	0.72	2.39	46	0.780				
	Au/Spiro/PVK/FTO	0.50	2.2	38	0.370				
Ag_2_BiI_5_	Au/Spiro/PVK/m‐TiO_2_/FTO	0.61	6.33	59	2.31	1.83	Hr 05% and 25 °C, retained PCE ≈100%	2018	^[^ ^132^ ^]^
Ag_3_BiBr_6_	Ag/P3HT/PVK/TiO_2_/FTO	0.60	0.12	45	0.033	2.58	Ambient, 7 days, retained PCE 95%	2019	^[^ ^160^ ^]^
Ag_3_BiI_6_	Au/PTAA/PVK/m‐TiO_2_/c‐TiO_2_/FTO	0.63	10.7	64	4.30	1.83	–	2017	^[^ ^17a^ ^]^
Cs_2_AgBiBr_6_	Au/Spiro/PVK/m‐TiO_2_/FTO	0.98	3.39	63	2.43	2.21	Ambient 1 sun illumination, 100 min, PCE retained ≈100%	2017	^[^ ^126^ ^]^
MA_3_Bi_2_I_10_	Ag/P3HT/PVK/TiO_2_/FTO	0.31	3.40	38	0.40	1.77	Ambient, 17 h, retained PCE ≈100%	2016	^[^ ^101^ ^]^
AgBi_2_I_7_	Au/P3HT/PVK/m‐TiO_2_/c‐TiO_2_	0.56	3.30	67	1.22	1.87	Ambient 10 days, retained PCE ≈100%	2016	^[^ ^130^ ^]^
HDABiI_5_	Ag/Spiro/PVK/c‐TiO_2_/m‐TiO_2_/FTO	0.40	0.115	43	0.027	2.10	200 °C, 60 min, no mass loss of the film, revealed by TAG analysis	2016	^[^ ^46^ ^]^
KBaTeBiO_6_	Pt/II_3_/PVK/TiO_2_/FTO	0.54	0.09	58	0.017	1.88	Ambient 380 days, film structure unchanged, revealed by XRD analysis	2019	^[^ ^129^ ^]^
(HDA^2+^)BiI_5_	Au/Spiro/Bi/m‐TiO_2_/ALD‐c‐TiO_2_/FTO	0.34	0.15	–	0.012	2.00	200 °C for 60 min, no mass loss of the powder, revealed by TAG analysis	2019	^[^ ^161^ ^]^
AgBiS_2_	Ag/MoO_3_/PTB7/PVK/ZnO/ITO	0.45	22.1	63	6.31	1.30	Ambient Several weeks, retained PCE ≈100%	2016	^[^ ^139^ ^]^

## 6 Other Applications

Due to their favorable photoabsorption, Bi‐based PVKs are mostly exploited for photovoltaics; although they have also been utilized for other optoelectronic applications, e.g., photodetectors, capacitors, and memory devices, which are discussed in the following section.

### 6.1 Photodetectors

**Figure 16:**
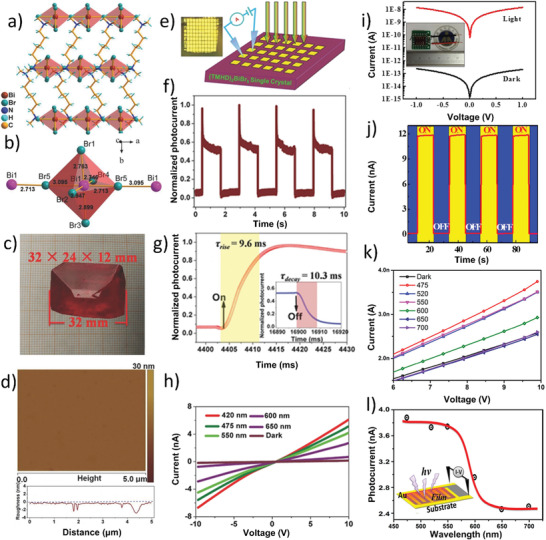
Single crystals and photodetecting device characteristics of Bi‐based perovskites: a–c) Crystal structure, structural unit and an optical image of the as‐grown single‐crystal of (TMHD)BiBr_5_. d) AFM image showing the surface roughness profile of the inch‐size (TMHD)BiBr_5_ single‐crystal. e) Schematics and optical image of the working photodetector device based on the oriented (TMHD)BiBr_5_ single‐crystal. f) Attenuation‐less time‐dependent photocurrent characteristics of the single‐crystal photodetector under chopped light irradiation measured for several cycles. g) Temporal photocurrent response; the highlighted regions are showing a rise and decay times 9.6 and 10.3 ms, respectively. h) *I*–*V* characteristics of the (TMHD)BiBr_5_ single‐crystal photodetector measured in dark as well as under light illuminations with various intensities and wavelengths. Reproduced with permission.^[^ ^143c^ ^]^ Copyright 2018, Wiley‐VCH. i) Typical *I*–*V* performance of the photodetector with Au electrodes both in light and in dark (plotted under log scale). The inset is a picture of the photodetecting device. j) Photoresponse of the red‐light photodetector when the 650 nm light was alternately turned on and off at +1 V bias voltage. Reproduced with permission.^[^ ^143b^ ^]^ Copyright 2017, American Chemical Society. k) Photocurrent response of the photodetecting device to monochromatic light in the visible range. l) Spectral photocurrent of the film device at different wavelengths (475–700 nm) under a bias of 10 V. Inset is a diagram photodetector under a xenon lamp light source with different filters. Reproduced with permission.^[^ ^143f^ ^]^ Copyright2018, American Chemical Society.

Photodetectors are PV devices that absorb photons and convert them instantly into electrical impulses. Photodetectors have got a key role in communications, remote sensing, biological and chemical sensing, computation, and imaging, etc. Due to higher absorption coefficients, Bi‐based PVKs can absorb light efficiently even if the film is a few hundred nanometers thick. Owing to confinement in such a small region, the charge carriers could not travel up to larger distances; therefore, the charge collecting electrodes should be closed enough to ensure faster carrier transport and photoresponse. Recently, Bi‐based PVKs were employed successfully for photodetection purposes.^[^ ^143^ ^]^ The first CsBi_3_I_10_‐based photodetector was fabricated in 2017 by Tong et al.,^[^ ^143b^ ^]^ see **Figure** **16**i,j. The device was quite sensitive to red light (650 nm) and showed a higher on/off ratio of 10^5^ along with a rapid response speed of *τ*_rise_ = 0.33 ms and *τ*_decay_ = 0.38 ms. The photodetector exhibited a specific detectivity of 21.8 A W^−1^ and photoresponsivity (ratio of current to incident light) of 1.93 × 10^13^ jones, which was superior to the Pb‐based photodetectors. Moreover, the device demonstrated a high external quantum efficiency of 4.13 × 10^3^% along with outstanding environmental stability for more than 3 months in the ambient air.

Similarly, a photodetector based on highly oriented single crystals of TMHDBiBr_5_ with dimensions of 32 × 24 × 12 mm^3^ was fabricated.^[^ ^143c^ ^]^ The photodetecting device showed an excellent performance with an on/off ratio of ≈10^3^ and a rapid response speed of *τ*_rise_ = 8.9 ms and *τ*_decay_ = 10.2 ms. Moreover, the photoresponsivity of the device was measured to be 100 mA W^−1^, which was almost invariant in the 365–700 nm range, see Figure 16a–h. The excellent photodetection performance of a device was attributed to the higher crystallinity, extended carrier lifetimes, and efficient carrier transport in the PVK architecture. Another visible light photodetecting device based on Cs_3_Bi_2_I_9_ nanoplates was fabricated by Qi et al.^[^ ^143d^ ^]^ Such a device was directly fabricated via facile solution processability on the ITO glass substrates and could also be deposited on any sort of substrate. This was the first photodetector based on CBIPVK with photoresponsivity of 33.1 mA W^−1^ under an illumination laser beam of 450 nm wavelength. The photoresponse of these devices was surprisingly 6‐times higher than the solution‐processed photodetectors based on MAPbI_3_ nanowires. Along with better operational stability, the specific detectivity of the device was observed to reach values as high as 10^10^ jones. Moreover, *τ*_rise_ and *τ*_decay_ were recorded to be 10.2 and 37.2 ms, respectively.

Recently, another photodetector based on vertically oriented single crystals of 0D PVK‐like material, i.e., (PD)_2_Bi_2_I_10_·2H_2_O was reported.^[^ ^143e^ ^]^ The photodetecting device exhibited relatively lower performance, i.e., a photocurrent of 194 mA with an on/off ratio of 2.1 and photoresponsivity of 1.14 mA W^−1^. In addition to this, the device also exhibited lower specific detectivity of 1.9 × 10^6^ jones and an external quantum yield of only 0.4%. Similar underperformed photodetectors were also fabricated from 1D and 2D PVK compounds, i.e., (C_6_H_13_N)_2_BiI_5_, (TMP)BiX_5_ (X = I, Br, or Cl), and 2D‐PVK, i.e., (TMP)_1.5_[Bi_2_I_7_Cl_2_],^[^ ^143f,g^ ^]^ see Figure 16k,l.

### 6.2 Memristors and Capacitors

**Figure 17:**
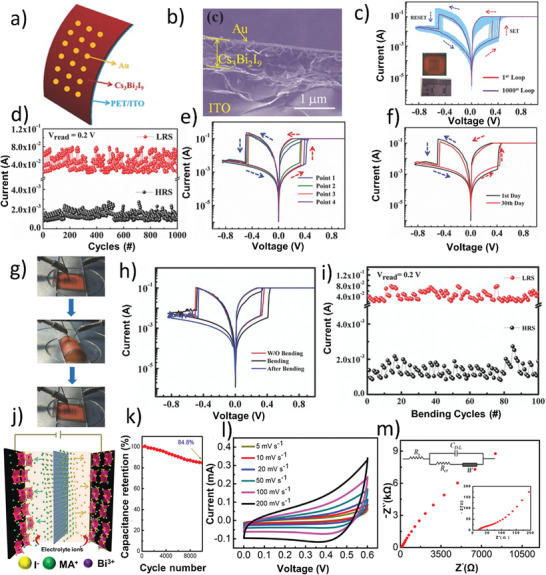
Resistive switching performance and stability of the Bi‐based resistive memory devices: a,b) Schematics and cross‐sectional SEM of the memristor device. c) Repeatability analysis of the bipolar memristor monitored by testing the *I*–*V* characteristics over 1000 cycles. d) Endurance of the memristor over 1000 cycles, where the high‐resistive state (HRS) and low resistive state (LRS) values were read at 0.2 V. e) *I*–*V* characteristics of the same memristor monitored at four different spots. f) Stability analysis of the device over 30 d. g) Photographs of the memristor during bending for a complete cycle. h) *I*−*V* characteristics without and during bending stress (bending radius = 0.9 cm). i) Endurance test of the memristor for 100 bending cycles, where HRS and LRS values were read at 0.2 V. Reproduced with permission.^[^ ^144^ ^]^ Copyright 2017, Wiley‐VCH. j) Schematics of the MBI‐based capacitor operation mechanism. k) A plot of retained capacitance with the number of cycles. l) *IV*‐characteristic of MBI‐based capacitor. m) The Nyquist plot, inset: equivalent circuit diagram. Reproduced with permission.^[^ ^146^ ^]^ Copyright 2017 American Chemical Society.

Other than the aforementioned optoelectronic applications, Bi‐based PVKs are also utilized for the resistive memory storage devices, i.e., memristors. The first flexible memristors based on the nanosheets of CBI and MBI were fabricated via the dissolution and recrystallization process.^[^ ^144^ ^]^ Such ultrathin memristors demonstrated a typical resistive‐switching behavior at a lower operating voltage of ≈0.3 V, remarkably higher ON/OF ratio ≈10^3^, and extended data retention of >10^4^ s. Additionally, the memory devices exhibited excellent environmental durability, flexibility, reproducibility, and endurability, see **Figure** **17**a–i. In that study, it was realized that due to the lack of long‐term stability, MBI nanosheets were found to be inappropriate for the fabrication of memristors. However, in another study, a solution‐processed memristor of MBI with a faster speed of 100 ns, reliable retention of ≈10^4^ s, and 300 cycles endurance was reported.^[^ ^145^ ^]^ The device showed a multilevel data storage capability with four resistive states and retained its properties for more than 5 months in the ambient environment.

Along with memristors, Bi‐based PVKs were also exploited for the fabrication of ultrafast capacitors. For instance, Pious et al.^[^ ^146^ ^]^ reported a double‐layered electrochemical capacitor of MBI thin film. A maximum capacitance of 5.5 mF cm^−2^ was demonstrated by the device and retained well up to 84.4% even after 10 000 charging–discharging cycles, see Figure 17j–m. Along with this, it was also revealed by the impedance spectroscopy that the active MBI layer provided a larger area for the electrolytes.

## 7 Opportunities and Challenges

Albeit the hallmarked Pb‐based PSCs have reached the summit of 25.2% PCE, its toxicity is still a major concern about its industrialization. Amongst the nontoxic alternatives, Bi‐based PSCs have shown promise, yielding outstanding environmental stability and optoelectronic properties, which are at par with the Pb‐based PVKs. After the pioneering work of Park et al.,^[^ ^55^ ^]^ numerous Bi‐based PVKs have been introduced and exploited for various optoelectronic and photovoltaic applications. However, the PCE of these compounds is on the lower side with a maximum of 6.3% reported for AgBiS_2_, while PCEs above 3% are rarely reported.

### 7.1 Enhancement of PCE in Bi‐Based PSCs

Lower PCE is attributed to a variety of reasons including, poor film morphology, indirect and wider bandgaps, bandgap offset between HTM and the active layer, and lower dimensionality. The challenge thereby is to address the root causes, in the forthcoming lines, three major causes and possible solutions are summarized.

**Figure 18:**
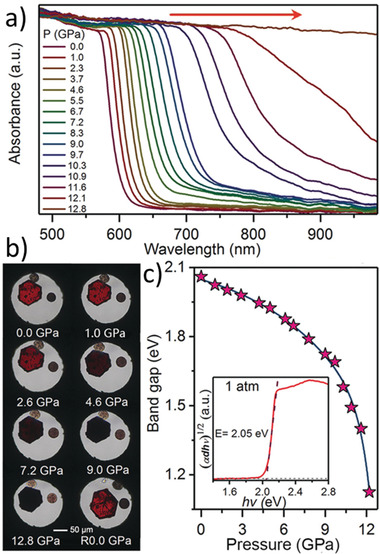
a) Optical absorption spectra of CBI under high applied pressure. b) Optical images of CBI in a diamond anvil cell (DAC) upon compression. c) Bandgap evolutions of CBI under high pressure. The inset of the figure shows a Tauc plot for CBI at 1 atm. Reproduced with permission.^[^ ^147^ ^]^ Copyrights 2018, Wiley‐VCH.

a) Improving film morphology: The quality of Bi‐based PVK films could be improved by pursuing the prevailing fabrication techniques, which are already utilized successfully for the Pb‐based PVK films. For instance, the morphology of the Bi‐based PVK films fabricated via a two‐step wet‐solution process was found to be superior to the ones prepared via a single‐step wet‐solution process. Likewise, the film quality was further improved when the dry and wet routes were combined. The film quality can be further improved with i) optimization of the precursor solution, ii) solvent‐engineering to decrease the volatility of the precursor solutions and their rapid crystallization, iii) use of antisolvent at the time of film fabrications, and iv) the introduction of the cost‐friendly novel solid‐state routes which have the potential for the fabrication of large‐area PVK films in the ambient environment.
b) Tuning the bandgap: It has been shown that external pressure can help to decrease the bandgap in the Bi‐based PVK crystals,^[^ ^147^ ^]^ see **Figure** **18**. The same strategy could be beneficial in the transformation from indirect to direct band structure.^[^ ^148^ ^]^ However, the exertion of such a high external pressure (i.e., 0–9.3 GPa) on the operating photovoltaic devices is not viable; therefore, an alternative way is to apply chemical pressure as previously done for the Pb‐based PVKs.^[^ ^149^ ^]^ Another strategy could be the fabrication of composites by the addition of materials with relatively lower bandgaps.^[^ ^139^ ^]^ Furthermore, the utilization of different experimental tools and theoretical calculations are required to explore the numerous possible combinations of the Bi‐based double PVKs, which could provide 3D Bi‐based PVK compounds with lower energy and direct bandgaps, suitable for optoelectronic applications.
c) Increasing the dimensionality: The lower dimensionality of Bi‐based PVKs (A_3_Bi_2_X_9_) is the main reason for their high exciton binding energy, shorter carrier lifetime, higher charge trapping density, and lower charge carrier mobilities. This issue could be addressed by Bi‐based double PVKs; unfortunately, out of their numerous combinations, only a few of them are explored yet. Detailed theoretical calculations are needed for the exploration of new double PVK compounds based on various combinations of M^+^ and M^3+^ with appropriate enthalpy, energy bandgap, exciton binding energy, and carrier effective masses. Additionally, it is reported that double PVKs show lower solubility in the conventional organic solvents; therefore, instead of borrowing the solvents from the Pb‐based PVKs, a hunt for new suitable solvents is vital.

Further enhancement in the efficiency can be achieved by designing novel device architectures different from the existing Pb‐based PSC. Owing to their comparatively deeper valence band maxima, there existed a larger bandgap mismatch between HTL and active PVK layer; for instance, between Cs_2_BiAgBr_6_ (6.04 eV) and spiro‐OMeTAD (5.13 eV).^[^ ^126^ ^]^ Therefore, novel hole‐transporting materials (HTMs) with minimum energy band offset are required to ensure better charge transport and high open‐circuit voltage. Moreover, the PCE could further be improved by a) the partial inclusion of Pb with Bi could fabricate synergetic PV devices with better performance and lower toxicity and b) surface passivation by utilizing suitable ligands.

### 7.2 Further Increasing the Stability

The following strategies can be used to further uplift the environmental stability of the Bi‐based PVKs: a) implanting inorganic or hydrophobic organic cations to improve their moisture stability, b) utilizing the metal nanoparticles to downconvert the high energy UV‐light into a visible lower energy radiations to improve their photostability, and c) employment of thermal‐resistive transparent 2D‐material on top of active material to increase the thermal stability of devices. Moreover, the stability could be improved further by surface modifications using certain additives; for example, ionic liquids and other moieties. Although the stability of the Bi‐based devices is reported to be better than the Pb‐based ones, still there is no detailed study been done to measure their stability in the controlled humidity, temperature, and light. Therefore, a standard testing procedure (e.g., a testing protocol provided by ISOS^[^ ^150^ ^]^) is required to analyze the exact stability of the Bi‐based PVKs.

### 7.3 Potential of Bi‐Based Perovskites

Bi‐based PVKs are nontoxic, highly stable, and possess excellent optoelectronic properties. The wider bandgap of phase‐pure undoped Bi‐based PVKs is nearer to the optimum bandgap of the top cell in Si‐based tandem cells (i.e., 1.7 eV for biterminal and 1.9 eV for four‐terminal devices), where Bi‐based alloyed PVKs exhibited a smaller bandgap closed to the optimum bandgap of the single‐junction solar‐cell.^[^ ^151^ ^]^ Due to thermal and photoinstability, the conventional Pb‐based PVKs cannot be used as a top layer in the tandem cells. Besides PV applications, Bi‐based PVK exhibited excellent performance (even better than Pb‐based PVKs) with extended stability in other optoelectronic applications; for instance, photodetectors, memristors, and capacitors. Unfortunately, there are only a few reports on such devices; therefore, extensive scrutiny is required to explore the potential of Bi‐based PVKs in optoelectronic applications other than solar‐cells. Similarly, there are few reports on the highly stable and more efficient inorganic Bi‐based perovskites (e.g., Cs_3_Bi_2_X_9_); therefore, a substantial effort is required for both material synthesis and device fabrication of the inorganic Bi‐based PVKs to utilize them for the optoelectronic applications. Moreover, the ambient environment processing of inorganic Bi‐based optoelectronic devices could significantly decrease the fabrication price.

Due to the aforementioned advantages, we conclude that Bi‐based PVKs have the potential to substitute the toxic Pb in various next‐generation optoelectronic devices. However, Bi‐based PVKs are still in the stage of material development and a collaborative multidisciplinary effort is required from researchers to understand their structural, optical, and electronic properties to effectively exploit them for various optoelectronic applications.

## Conflict of Interest

The authors declare no conflict of interest.
